# Optimized Extraction of Polyphenols from Unconventional Edible Plants: LC-MS/MS Profiling of Polyphenols, Biological Functions, Molecular Docking, and Pharmacokinetics Study

**DOI:** 10.3390/molecules28186703

**Published:** 2023-09-19

**Authors:** Hafiza Sehrish Kiani, Waheed Ahmad, Sana Nawaz, Mohammad Abul Farah, Akhtar Ali

**Affiliations:** 1 Department of Microbiology, Quaid-i-Azam University, Islamabad 45320, Pakistan; sehrishkiani30@gmail.com; 2 State Key Laboratory of Bioreactor Engineering, East China University of Science and Technology, Shanghai 200237, China; 3 Department of Nutritional Sciences, Government College University Faisalabad, Faisalabad 38000, Pakistan; sananawaz388@gmail.com; 4 Department of Zoology, College of Science, King Saud University, Riyadh 11451, Saudi Arabia; mfarah@ksu.edu.sa; 5 School of Agriculture, Food and Ecosystem Sciences, Faculty of Science, The University of Melbourne, Parkville 3010, Australia

**Keywords:** medicinal plants, polyphenols, flavonoids, chicory, ryegrass, moringa, lemongrass, human health, diabetes

## Abstract

Plant bioactive phenolic metabolites have recently attracted the attention of researchers due to their numerous health advantages. Therefore, this study aimed to investigate with advanced techniques the bioactive metabolites and antioxidant and antidiabetic capacity of four unconventional edible plant leaves: lemongrass (*Cymbopogon citratus* (DC.) Stapf), chicory (*Cichorium intybus* L.), moringa (*Moringa oleifera* Lam.), and ryegrass (*Lolium perenne* L.). The extraction process was optimized using different solvents. These plants’ phenolic composition, identification, and characterization have been determined herein using LCESI-QTOF-MS/MS. This research identified 85 phenolic compounds, including 24 phenolic acids, 31 flavonoids, 7 stilbenes and lignans, and 17 other metabolites. Moreover, the study determined that moringa has the highest total phenolic content (TPC; 18.5 ± 1.01 mg GAE/g), whereas ryegrass has the lowest (3.54 ± 0.08 mg GAE/g) among the selected plants. It seems that, compared to other plants, moringa was found to have the highest antioxidant potential and antidiabetic potential. In addition, twenty-two phenolic compounds were quantified in these chosen edible plants. Rosmarinic acid, chlorogenic acid, chicoric acid, ferulic acid, protocatechuic acid, and caffeic acid were the most abundant phenolic acids. In silico molecular docking was also conducted to investigate the structure–function relationship of phenolic compounds to inhibit the alpha-glucosidase. Finally, the simulated pharmacokinetic characteristics of the most common substances were also predicted. In short, this investigation opens the way for further study into these plants’ pharmaceutical and dietary potential.

## 1. Introduction

For thousands of years, people have utilized plants to improve their health. Until the 19th century, traditional plants were used for therapeutic purposes [1]. For example, herbal tea from plants treats digestive issues [2]. Traditional phytotherapy uses plants for medicinal purposes, and it has long been a choice for historical and modern treatment [3]. More than 80% of the population, particularly in underdeveloped nations, benefits from herbal products for medicinal purposes. Nowadays, it is a significant field of study to develop plant medications in addition to the traditional use of plants for treatments.

The phytochemicals found in medicinal plants enhance the control and prevention of metabolic diseases. These compounds have an additive and synergistic therapeutic impact [4,5]. Medicinal plants provide a variety of biologically active substances that have positive health effects. Phytochemicals from medicinal plants involve numerous complex biochemical, metabolic, and physiological mechanisms and have been studied for their impact on human health and wellness. Moringa and lemongrass are well-known plants due to their therapeutic potential. Moringa (*Moringa oleifera*) is used in herbal medicine and, as fresh leaves, a flavoring agent in many foods and beverages, and possesses cytotoxic and antibacterial properties [6]. Moringa is also a good source of antioxidant metabolites [7], used for gastric acidity treatment [8,9] aerophobia [10,11], nausea [12,13] vomiting, pharyngitis, liver issues, anorexia, stomach aches, etc. [14,15]. In vitro and animal studies indicate that moringa leaves may have more medical applications than are known at present. Studies have revealed that moringa has antibacterial, stress-relieving, and anticancer properties. The most well-known benefit of moringa is as a digestive aid. Irritable bowel syndrome-related stomach pain may be relieved by moringa oil, based on a brief study. There is sufficient evidence from pharmacological studies to support the biological effects of moringa and the phytochemicals found in the plant [16]. Additionally, mouth, throat, and sore throat irritation are also treated with it. Moringa (*Moringa oleifera*) is a nutrient-rich plant with various health benefits. It contains bioactive compounds like flavonoids, polyphenols, and alkaloids that exhibit antidiabetic properties. Moringa may help lower blood sugar levels by improving insulin secretion and reducing insulin resistance [17]. Additionally, its antioxidant and anti-inflammatory properties may play a role in mitigating diabetic complications.

Lemongrass (*Cymbopogon citratus*) contains compounds such as citral and polyphenols that have shown potential antidiabetic effects. Research suggests that lemongrass may help lower blood sugar levels by enhancing insulin sensitivity and improving glucose metabolism. Its antioxidant properties can also contribute to reducing oxidative stress, a factor linked to diabetes complications [18]. Further, lemongrass leaves have a strong, enticing, lemony scent and have been used for both culinary and medicinal purposes for many years. Traditionally, several illnesses and medical disorders have been treated with infusions produced from the plant’s fresh or dried leaves. These can include stomach issues, nervous system disorders, cough, rheumatism, nausea, and vomiting, as well as muscle and joint pain. In addition to having mild natural analgesic and urinary stimulant effects, research has revealed that the plant also contains anticancer, antioxidant, and anti-inflammatory properties [19,20]. As for protecting the stomach lining, lemongrass tea has been known to relieve gastrointestinal irritations, indigestion, and ulcers in the gastric area [21]. 

Unconventional edible plants may exhibit potential antioxidant and antidiabetic activities. Chicory (*Cichorium intybus*) is a plant whose roots are often roasted and used as a coffee substitute. It contains inulin, a type of soluble fiber that acts as a prebiotic, supporting gut health. Inulin can help regulate blood sugar levels by slowing the absorption of glucose in the digestive tract. Studies have indicated that chicory may have hypoglycemic effects and could potentially be beneficial for managing diabetes [22]. Chicory has several vital substances that are significant from a medicinal perspective. These substances include vitamins, chlorophyll, flavonoids, inulin, tannins, alkaloids, unsaturated sterols, saponins, and coumarins [23,24,25]. One of the most efficient ways to manage and lessen the consequences of chronic diseases like diabetes mellitus is to consume chicory, which is a rich source of terpenoids and phenolic compounds. Chicory plants also have therapeutic qualities such as antioxidant activity, usage for wound healing, assistance with diabetes, and the ability to fight harmful pathogens [26,27]. 

Ryegrass (*Lolium perenne*) is known for its high fiber content and slow digestion rate. The soluble and insoluble fibers in ryegrass contribute to improved glycemic control by slowing the release of glucose into the bloodstream. This can help prevent rapid spikes in blood sugar levels after meals. Ryegrass consumption has been associated with reduced risk of type 2 diabetes and improved insulin sensitivity. The ryegrass plant is an unconventional food plant that has a significant quantity of bioactive substances, including protein, fatty acids, alkylresorcinols, folates, tocotrienols, tocopherol, lignan, phytosterols, and phenolic acid [28,29,30]. In addition to helping with weight loss and appetite control, ryegrass consumption also lowers the risk of cancer, type 2 diabetes, cardiovascular disease (CVD), and other chronic diseases, along with mitigating allergic and inflammatory reactions [31,32].

Moreover, unconventional edible plants are excellent sources of antioxidants for preserving food and beverages. Numerous phenolic compounds that act as antioxidants, anticarcinogens, antimutagens, or anti-inflammatory agents are being studied for their potential to prevent cancer. Green edible plants are used for enhancing the organoleptic qualities (aroma, flavor, and taste) of many food products, while their possible health-promoting effects receive less attention [33]. The primary phytochemicals in these plants are polyphenols. Functional and therapeutic foods are in higher demand due to consumer demand for goods with possible health advantages. The most popular unconventional edible plants, moringa and lemongrass, have a variety of uses because they contain bioactive compounds that are employed in pharmacy, skincare products, and medications, in addition to their use as supplementary food [34]. To increase the effectiveness of medicinal effects, the synergistic effects of nontraditional edible plants were examined. It is true that integrating them into a single product can be done by mixing their complementary phenolic compositions, which may bring up synergic effects for edible plants in boosting quality and potential health impacts of products.

A few studies have been conducted previously using single LC-MS [35,36] and without mass spectrometry [37,38,39] or MS/MS with limited profiling of complex mixture of plant extracts [40]. Still, complex profiling using LC-MS/MS is lacking due to the complex structure of phytochemicals and unavailability of pure standards. LC-ESI-QTOF-MS/MS is equipment that provides a more reliable, sensitive, and authentic untargeted profile of phytochemicals from a complex mixture of plant extracts. To achieve this, samples were extracted using different solvents to analyze the profile of phenolic components and antioxidant activity. Phenolic compounds were assessed using LC-ESI-QTOF-MS/MS (electrospray ionization–quadrupole time-of-flight mass spectrometry). To estimate the total phenolic compounds and antioxidant potential, other methods include the total phenolic contents (TPC), total flavonoid contents (TFC), total condensed tannins (TCT), 2,2-azinobis-3-ethylbenzothiazoline-6-sulfonic acid (ABTS), hydroxyl radical scavenging assay (OH-RSA), DPPH, and FICA were used. The thorough profile of phenolics and their antioxidant and antidiabetic activities are examined for the first time in this group of edible plants. Additionally, using the pkCSM and SwissADME platforms, their predicted absorption, distribution, metabolism, excretion, and toxicity models were investigated. In silico molecular docking was also conducted to understand the structure–function relationship of phenolic compounds in these edible plants. This study demonstrates the potential and significance of medicinal and edible plants as a potential source of bioactive metabolites in various business sectors, including functional foods, skincare products, pharmaceutical and therapeutic products, and even the livestock sector.

## 2. Results and Discussions

### 2.1. Extraction Process Optimization for Total Phenolic Content in Selected Unconventional Edible Plants

The extraction process to extract total phenols from selected unconventional edible plants was optimized using different solvents. The results of different solvents extraction are given in Table 1. 

Six different solvent systems such as methanol (80%), ethanol (80%), acidified methanol (80%), acidified ethanol (80%), acetone (80%), acidified acetone (80%), and water were used for the extraction process. In terms of 80% methanol, maximum extraction of total phenolic content in moringa was observed to be 18.0 ± 1.51 mg GAE/g, whereas chicory exhibited its minimum extractions of total phenolic content, i.e., 4.76 ± 0.11 mg GAE/g. Previously, an analysis of the methanolic extract revealed that the phenolic content in the moringa leaf extract was substantially (*p* ≤ 0.001) higher than that in the floral extract. The amount of phenols in moringa leaf extract was 111% higher than that in its flower extract. A leaf of *M. oleifera* Lam had 2.28 mg/mL total phenols, whereas a floral extract of the same plants contained 1.08 mg/mL total phenols [41]. Utilizing 80% ethanol as the solvent, the greatest yield of total phenolic content from moringa was measured at 16.2 ± 1.33 mg GAE/g, whereas the lowest total phenolic content extraction was recorded at 3.12 ± 0.02 mg GAE/g. The highest yield of total phenolic content from moringa, recorded at 18.5 ± 1.01, was achieved using 80% acidified methanol as the solvent. In contrast, the lowest recovery of total phenolic content, measuring 3.54 ± 0.03, was obtained in ryegrass when 80% acidified methanol was employed. The peak extraction of total phenolic content from moringa, reaching 17.1 ± 1.32 mg GAE/g, was obtained when utilizing 80% acidified ethanol, while the least total phenolic extraction was observed with acidified ethanol, at 3.43 ± 0.09 mg GAE/g. Vania Urías-Orona et al. also proposed the effect of the extraction solvent on the phenolic content in a commercial dietary supplement made from moringa oleifera leaves. According to their study, methanol (MeOH) and ethanol (EtOH) at concentrations of 100%, 80%, and 50% aqueous were used as extraction solvents. The amounts of total phenols collected varied between 55.98 and 226.20 mg ChlAE/g, with EtOH 50% and EtOH 100% having the highest and lowest contents, respectively. There were significant variations in the phenolics analyses (*p* < 0.05) [42]. Moringa’s total phenolic content was best extracted using 80% acetone and 80% acidified acetone, yielding 16.1 ± 1.09 mg GAE/g and 17.2 ± 1.47 mg GAE/g, respectively. On the other end, the least efficient extraction of total phenolic content in ryegrass, using 80% acetone and 80% acidified acetone, resulted in 1.84 ± 0.01 and 1.63 ± 0.02, respectively (Table 1). The phenolic activity in ryegrass following solvent extraction was discovered by Darius Povilaitis et al. in their work. The TPC values for ryegrass ranged widely (12.50.19–530.1 mg GAE/g), with acetone extracts having the highest TPC (up to 530.11 mg GAE/g), followed by hexane and then methanol extracts. As a result, the most effective solvent for extracting phenolic compounds from ryegrass was acetone [43]. Overall, 80% acidified methanol provided better extraction than other solvent mixtures. 

### 2.2. Quantification of Total Polyphenols in Selected Plant Extracts

Based on the optimization results, we proceeded with 80% acidified methanol extraction for biological activities and LC-MS/MS analysis. Researchers have been very interested in phenolic compounds since a range of phenolic compounds are examined that have chemoprotective abilities, which function as antioxidant, anticarcinogenic, antimutagenic, or anti-inflammatory chemicals. Table 2 lists the phenolic concentrations in selected plants. 

The Folin–Ciocalteu technique was used to determine the total phenolic content (TPC). The findings revealed that the phenolic content in moringa, measured at 18.5 ± 1.01 mg GAE/g, was higher than that of lemongrass, measured at 12.9 ± 0.12 mg GAE/g, chicory measured at 4.85 ± 0.06 mg GAE/g. The lowest measured was in the ryegrass sample, at 3.54 ± 0.08 mg GAE/g. Previously, a higher amount of TPC was measured in moringa at 103.28 ± 8.08 mg GAE/g than that of the other investigated samples [44]. The TPC (12.9 ± 0.12 mg GAE/g) in our study is lower than the TPC of Australian lemongrass (15.09 ± 0.88 mg QE/g) [20]. The TPC results might vary because different samples were used in the current study and other studies to assess the phenolic levels. 

According to an analysis of total flavonoid content, moringas had a much higher quantity of flavonoid content (10.1 ± 0.83 mg QE/g) than lemongrass (6.19 ± 0.09 mg QE/g) and chicory (1.06 ± 0.01 mg QE/g). Findings show that ryegrass (1.52 ± 0.13 mg QE/g) has the lowest concentration of flavonoids among the selected group of plants. Flavonoids are the most common class of secondary plant metabolites and are employed in pharmaceutical, medical, and cosmetic applications because of their antioxidative, anti-inflammatory, anticarcinogenic, and antimutagenic properties [45]. In a similar study, the flavonoid content in ryegrass was investigated, and the same results of TFC were reported for ryegrass, revealing 0.01 ± 0.01 mg QE/g [36]. 

Chicory showed significantly higher levels of total tannin content (1.31 ± 0.15 mg CE/g) compared to other selected edible plants. Previous investigations on the measurement of total tannin contents found similar results in Australian lemongrass (1.36 ± 0.08 mg CE/g) [20], moringa (8.31 ± 1.58 mg CE/g) [44], ryegrass (2.11 ± 0.06 mg CE/g) [36], and chicory (0.84 ± 0.03 mg CE/g) [35]. Moringa is a suitable natural antioxidant or a useful ingredient in food, cosmetics, and pharmaceuticals due to its overall much greater phenolic chemical profile. Owing to their potential health advantages, flavonoids, the most abundant class of phytochemicals found in plants, fruits, and medicinal plants, have attracted much attention. These medicinal plants were never combined in previous research. These plants’ flavonoids and total phenolic contents vary significantly from each other. Overall, moringa contains the highest amount of total phenolic and total flavonoids than other studied unconventional edible plants.

### 2.3. Antioxidant and Antidiabetic Activities of Selected Plant Extracts

For the various kinds of activities that were analyzed, such as radical oxygen scavengers, metal chelators, reducing agents, and H^+^ ion donators, antioxidants are recognized as essential components. Several assays, including the DPPH, ABTS, ferrous ion chelating activity (FICA), and ^•^OH-RSA, were conducted to evaluate the antioxidant capacity of herbal plants. Table 3 shows the results of antioxidant and antidiabetic potential for selected samples (moringa, lemongrass, chicory, and ryegrass). 

The cost-effective DPPH test is usually performed to estimate the samples’ capacity to neutralize free radicals in biological systems, which is based on their capacity to release electrons or hydrogen ions. DPPH, a free radical with a stable nitrogen center, loses part of its bluish-purple pigments when mixed with sample extracts [46]. When investigated for its ability to scavenge DPPH radicals, moringa (34.16 ± 2.32 mg AAE/g) showed considerably (*p* ≤ 0.05) greater antioxidant capacity than lemongrass (25.73 ± 0.18 mg AAE/g), chicory (16.61 ± 0.23 mg AAE/g), or ryegrass (11.18 ± 0.23 mg AAE/g). Similar radical-scavenging capacities in ryegrass (0.05 ± 0.01 mg AAE/g) were found in previous studies [36] on the antioxidant activity and total polyphenol content in a variety of unconventional dietary plants. 

The findings of the ABTS test revealed that the activities in ryegrass (18.4 ± 0.56 mg AAE/g) were significantly (*p* ≤ 0.05) lower than those in other green plants (moringa 54.9 ± 4.24, lemongrass 44.81 ± 0.93, and chicory 35.4 ± 0.68 mg AAE/g). Additionally, a ferrous ion chelating assay was carried out to determine the antioxidant capacity of the sample extracts, and the results showed that moringa had a substantially greater potential than other green plants (7.49 ± 0.39 mg EDTA/g) (*p ≤* 0.05). Previous research has also shown [44] the same results. Furthermore, hydroxyl radical scavenging activity was carried out to estimate the antioxidant profile, and the findings showed that moringa had greater activities. Overall, the findings in this study for all antioxidant assays indicated that moringa had the highest antioxidant activity and that ryegrass plants had the lowest antioxidant activity. 

The alpha-glucosidase IC_50_ (μg/mL) value is the inhibitory concentration needed to block 50% of the enzyme’s activity of the α-glucosidase enzyme. The enzyme activity is more effectively inhibited by the inhibitor when the IC_50_ value is lower. In this current study, comparing the inhibitory activity in the four edible plants selected (lemongrass, chicory, ryegrass, and moringa) against α-glucosidase, our results revealed that compared among all selected plant extracts, ryegrass showed lower enzyme inhibition activity, with IC_50_ = 29.02 ± 2.17 µg/mL. Ryegrass was followed by chicory, which also exhibited moderate inhibitory activity against αglucosidase, with IC_50_ values of 16.41 ± 1.21 µg/mL. The IC_50_ for α-glucosidase inhibitory activities of lemongrass and moringa were 2.15 ± 0.13 and 1.89 ± 0.01 µg/mL, respectively, which are considered as strong. In terms of α-glucosidase inhibitory effects, Shiva Nouri et al. identified *Moringa peregrina* (MP) and *Ferulago carduchorum* (FC) as two promising plants [47]. The regression equation representing the percentage of enzyme inhibition at various doses was used to estimate the IC_50_ values for MP and FC extracts. An increased enzyme inhibitory activity is indicated by a lower IC_50_ value. Their findings showed that the IC_50_ for MP was 4.96 μg/mL and the IC_50_ for FC was 7.41 μg/mL. The MP extract found to be more effective at inhibiting α-glucosidase [47]. 

### 2.4. Correlation Analysis

Correlation analysis was performed between the phenolic content (TPC, TCT, and TFC) of the chosen samples and the antioxidant activity produced by the four separate assays (Table 4).

The TPC had a significant positive correlation with TCT (*r^2^* = 0.99), DPPH (*r^2^* = 0.99), FICA (*r^2^* = 0.93), ^•^OH-RSA (*r^2^* = 0.99), and ABTS (*r^2^* = 0.93); the TFC had a positive correlation (*p* ≤ 0.1) with the all four antioxidant assays DPPH, ^•^OH-RSA, ABTS, and FICA. This seems to demonstrate a direct connection between the samples’ phenolic components and the antioxidant processes of ferric chelation, peroxyl inhibition, and free radical scavenging. 

**Figure 1:**
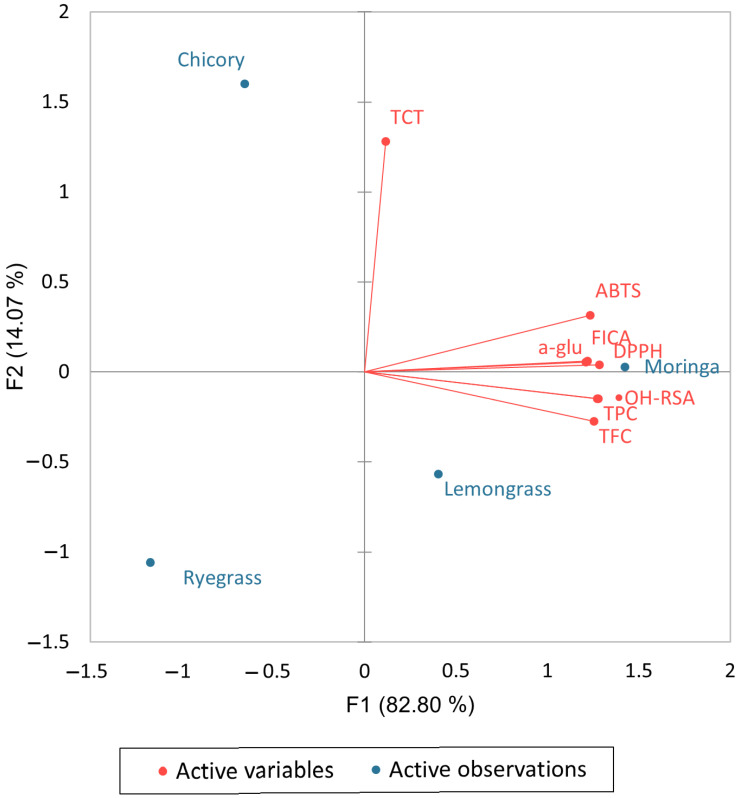
A biplot analysis of phenolic contents and biological activities of selected edible plants.

Flavonoids showed significant hydroxyl inhibition, free radical scavenging, and ferric ion chelation activity correlations. This further demonstrates the variety of phenolic and nonphenolic metabolites found in plant extracts. This may be related to the fact that a flavonoid’s capacity to act as an antioxidant is affected by the location of the hydroxyl group on the B-ring and whether it can deliver a free radical, either a hydrogen atom or an electron, or both. A biplot analysis (Figure 1) was conducted to investigate the correlation between active variables (TPC, TFC, TCT, DPPH, ABTS, FICA, ^•^OH-RSA, and alpha-glucosidase inhibition activity) and active observations (lemongrass, chicory, ryegrass, and moringa). 

The interactions between antioxidant activity and phenolic compounds might also be affected by the conditions of the study, the mechanism behind the antioxidant reactions, and the collaborative or antagonistic effects of other compounds present in the reaction mixture.

### 2.5. Characterization of Phenolic Compounds Using LC-MS

Green herbaceous plants have currently gained the attention of scientists due to the discovery that they contain significant amounts of bioactive components, specifically phenolic metabolites, which have demonstrated potential health benefits in numerous studies [44,48,49]. Using the latest method of LC-ESI-QTOF-MS2, phenolic compounds from the targeted samples of moringa, lemongrass, chicory, and ryegrass plants were screened and characterized. We can identify all the bioactive components of plant samples with an advanced analytical approach. The compounds were qualitatively characterized using Agilent Mass Hunter software, version B.06.00, and Personal Compound Database Library (PCDA). Table 5 lists the results associated with the phenolic chemical profile of plants. A total of 85 phenolic compounds have been found by LC/MS analysis, including 24 phenolic acids, 31 flavonoids, 6 isoflavonoids, 7 stilbenes and lignans, and 17 other polyphenols. The base peak chromatograms (BPC) of the selected plants and MS/MS spectra of some selected compounds are given in Figures S1 and S2.

#### 2.5.1. Phenolic Acids

Secondary plant metabolites called phenolic acids are well known for their potential health advantages. Owing to their potential for antiaging, antioxidant, antibacterial, anticancer, cardioprotective, anticancer, and anti-inflammatory activities, they are widely used in many products in the food, cosmetic, and pharmaceutical industries. The samples of green plants included a total of 24 phenolic acids, primarily hydroxycinnamic acid molecules. When CO_2_ and the hexosyl moiety from the parent ions are removed, these phenolic acids show a fragmentation pattern. Gallic acid (*m*/*z* 171.0298), protocatechuic acid (*m*/*z* 155.0339), and *p*-hydroxybenzoic acid (*m*/*z* 137.0244) were recognized as the compounds **1**, **2**, and **4** are benzoic acid derivatives, and they each displayed the product ions at *m*/*z* 109, 153, and 93 that existed in all samples, respectively. Another study [35] that used the LC-MS/MS approach to identify hydroxybenzoic acids and hydroxycinnamic acids in various Australian plants also found similar metabolites. Other hydroxybenzoic acids included protocatechuic acid 4-O glucoside (*m*/*z* 315.0721), which was only identified in two plants (lemongrass and moringa).

Twenty hydroxycinnamic acids were found. Only compound **7**, a cinnamic acid derivative identified as 3-caffeoylquinic acid (*m*/*z* 353.0878) with product ions at *m*/*z* 253, 190, and 144, was found in all four samples. Compounds **5**, **11**, **12**, **15**, **20**, and **21**, all found in moringa, lemongrass, and chicory samples, were identified as 3-sinapoylquinic acid (*m*/*z* 399.1286), caffeic acid (*m*/*z* 179.035), 3-feruloylquinic acid (*m*/*z* 367.1034), p-coumaroyl glycolic acid (*m*/*z* 221.0455), cinnamic acid (*m*/*z* 147.0451) and *p*-coumaric acid (*m*/*z* 163.04). Compounds **11** and **12** produced ions at *m*/*z* 143, 135, 133, 298, and 288 together with 192 and 191. Compounds **20** and **21** have product ions at *m*/*z* 129, 103, and 119, respectively. Only lemongrass and ryegrass samples were analyzed for the product ions of compounds **10** and **17**, which were identified as 1-*O*-sinapoyl-beta-D-glucose (*m*/*z* 385.114) and 1,2-diferuloylgentiobiose (*m*/*z* 693.2036), and showed ions at *m*/*z* 223, *m*/*z* 193, and *m*/*z* 134 after removing glucoside moiety (162 Da), water (18 Da), and CO_2_ (44 Da) from the precursor ion (*m*/*z* 385.1140), and then from product ions. *p*-Coumaroylquinic acid (*m*/*z* 337.0929), sinapic acid (*m*/*z* 223.0612), p and 3,5-diferuloylquinic acid (*m*/*z* 543.1508) were found to produce ions at *m*/*z* 193, *m*/*z* 191, *m*/*z* 179, *m*/*z* 149, and *m*/*z* 134, in contrast to compounds **13**, **14**, and **23**, which were only found in lemongrass and moringa samples. Similar findings were reported during previous research [20,35] that identified polyphenols in several Australian plants and plants. Their findings reveal many hydroxycinnamic acids, some of which resembled the compounds found in the current research.

#### 2.5.2. Flavonoids

Flavonoids are the most prevalent category of plant metabolites that are used in medical, beauty, and pharmacological industries for their antioxidative, anti-inflammatory, anticarcinogenic, and antimutagenic qualities [45]. In the current study, a total of 45 flavonoids were identified. Flavonoids were observed higher in amount than all the other phenolic compounds from the targeted samples. Observed categories of flavonoids included 4 flavanols, 4 flavanones, 7 flavones, 11 flavonols, 8 Isoflavonoids and others.

Theaflavin 3-*O*-gallate (*m*/*z* 715.1304) was the first observed flavanol, as compound **25**, in negative mode and produced product ions at *m*/*z* 565 and 139, only identified in the lemongrass and ryegrass samples. No flavanol compounds were found in all four samples. In addition, two compounds were only identified in chicory and moringa: procyanidin trimer C1 and epigallocatechin 3-*O*-gallate-forming product ions at *m*/*z* 739, 695, 577, and 451 for compound **30**, and 305 and 169 for compound **32**. A similar (−)-epicatechin compound was observed in the lemongrass, moringa, and chicory samples in both modes, and previous results revealed the presence of similar compounds in lemongrass and moringa samples.

##### Flavonols

Compound **47** was observed as quercetin 3-*O*-xylosyl-glucuronide, forming product ions at *m*/*z* 301 in negative mode only, identified in the ryegrass plant source, while three flavanol compounds that were only identified in lemongrass were kaempferol 3-O-xylosyl-glucoside, kaempferide, and isorhamnetin 3-*O*-rutinoside. Compounds **49**, **52**, and **54** were revealed by their product ions at *m*/*z* 285, *m*/*z* 283, *m*/*z* 151, and *m*/*z* 315, respectively. Myricetin 3-*O*-rhamnoside and 3,7-dimethylquercetin were identified only in lemongrass and moringa.

#### 2.5.3. Isoflavonoids

In this study, six Isoflavonoids were observed in the given samples. Compounds **56**, **59**, and **60**, identified as 6″-*O*-malonylglycitin, dihydroformononetin, and glycitein 7-*O*-glucuronide, were only present in lemongrass and moringa samples, as revealed their product ions at *m*/*z* 533, *m*/*z* 253, *m*/*z* 137, *m*/*z* 441, *m*/*z* 283, and *m*/*z* 267. Daidzein 7-*O*-glucuronide was only identified in lemongrass and chicory, while 6″-*O*-malonylgenistin was only identified in moringa and chicory samples. An isoflavonoid was only identified from samples of moringa, referred to as equol 7-*O*-glucuronide (*m*/*z* 417.1191), and forming its ions on *m*/*z* 241.

#### 2.5.4. Stilbenes and Lignans

The current study observed two stilbene and five lignan compounds from four studied samples. Compound **62** was identified as piceatannol (*m*/*z* 243.0663) and only found in moringa and chicory, while compound **63** was identified as 3′-hydroxy-3,4,5,4′-tetramethoxystilbene (*m*/*z* 303.1227), only observed in lemongrass and moringa samples. Numerous studies have been conducted on piceatannol, a stilbene with two phenol rings that was found in moringa and chicory, and has considerable health benefits including antioxidant, anticancer, antimutagenic, antiatherosclerotic, and anti-inflammatory properties [50,51]. LC-MS characterized bioactive substances, including lignans, which exhibit exceptional antioxidant and anticarcinogenic activities. Human ovarian, breast, and prostate cancers are protected or prevented from spreading by consuming plant lignans [52]. LC-MS helped to identify five lignans from the selected variety of samples. Compound **64**, medioresinol (*m*/*z* 389.1595), was only identified in lemongrass and moringa when observed in positive mode, while compound **65**, lariciresinol-sesquilignan (*m*/*z* 555.2235), was observed in both modes and found in only the lemongrass, chicory, and moringa samples. Other three lignans that were identified in negative mode were secoisolariciresinol-sesquilignan (*m*/*z* 557.2392), lariciresinol (*m*/*z* 359.15), and matairesinol (*m*/*z* 357.1343), and found only in the moringa sample. Another study [53] conducted on phytochemicals with cancer-fighting properties revealed some compounds observed from the lignan profile that were similar to the compounds identified in the current research.

#### 2.5.5. Other Compounds

A few coumarin derivatives were identified from all samples, four of which were only observed in lemongrass and moringa samples. These compounds identify as coumarin (*m*/*z* 145.0295), esculin (*m*/*z* 339.0721), umbelliferone (*m*/*z* 161.0244), and mellein (*m*/*z* 177.0557) as revealed by their product ions at *m*/*z* 101, *m*/*z* 177, and *m*/*z* 133, respectively. Three phenolic compounds characterized from the moringa sample were only identified as rosmanol, rosmadial, and hydroxytyrosol 4-O-glucoside, forming product ions at *m*/*z* 301, *m*/*z* 299, *m*/*z* 153, and *m*/*z* 123, respectively. Other terpene molecules found only in lemongrass and moringa were carvacrol (compound **70**) and carnosol (compound **72**), observed to be fragmenting at *m*/*z* 107 and *m*/*z* 285, respectively. 

Using LC-ESI-QTOF-MS/MS, we were able to screen and characterize 85 polyphenolic compounds with their product ions in samples of plants. All of these plant’s phenolic constituents have not yet been the subject of a single study. Screening for these bioactive components in these plants can provide a new field of study toward revealing their significant health advantages and new applications. The use of this sophisticated analytical method has significant promise for the discovery of novel, undiscovered bioactive chemicals. Low collision energies have the drawback of inability to locate the position of the native phenolic ring in the analysis of LC-MS/MS that underwent alteration. 

### 2.6. Venn Distribution of Polyphenols in Plant Extracts

**Figure 2:**
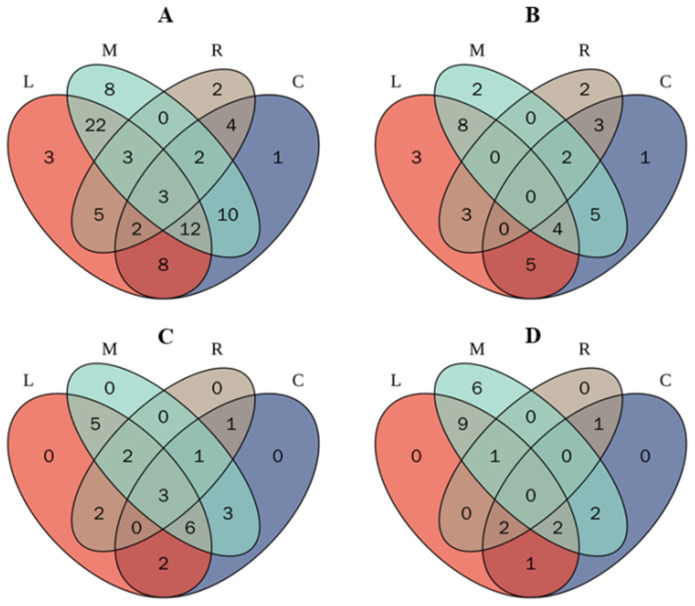
Distribution of phenolic compounds (Venn diagram) in lemongrass (L), moringa (M), ryegrass (R), and chicory (C): total phenolic (**A**), flavonoids (**B**), phenolic acids (**C**), and other compounds (**D**).

The Venn diagram is a practical, potent, and adaptive tool that can rapidly examine a large number of facts and transform it into information that is easy to understand. The Venn diagram illustrates the distribution of phenolic metabolites in lemongrass, moringa, ryegrass and chicory (Figure 2).

According to the Venn diagram (Figure 2A), three distinct phenolic compounds were found in the lemongrass sample, whereas moringa, chicory, and ryegrass, respectively, contained 8, 2, and 1 distinctive phenolic compounds. This shows that moringa possesses a wider variety of phenolic metabolites than other herbaceous extracts, which may help explain its increased TPC, TFC, and antioxidant potential (Table 1). It is interesting to see that moringa and lemongrass have the highest number of overlapping phenolic compounds. The total amount of flavonoid compounds in these edible plants is shown in a Venn diagram (Figure 2B). Compared to ryegrass and chicory, which have fewer distinct flavonoids, it was found that lemongrass and moringa have a wider diversity of unique flavonoids. 

This figure also shows that there are eight flavonoid components that are identical in lemongrass and moringa, but no single flavonoid touches all four sources. The total phenolic acids in the chosen plants are shown in Figure 2C. It demonstrates that moringa contains the most phenolic acids. Chicory also contains sixteen phenolic acids. Moringa contains a total of sixteen phenolic compounds (Figure 2D). None of the other phenolic metabolites were found to overlap in ryegrass, moringa, or chicory. 

### 2.7. Quantification/Semiquantification of Selected Phenolic Compounds

**Figure 3:**
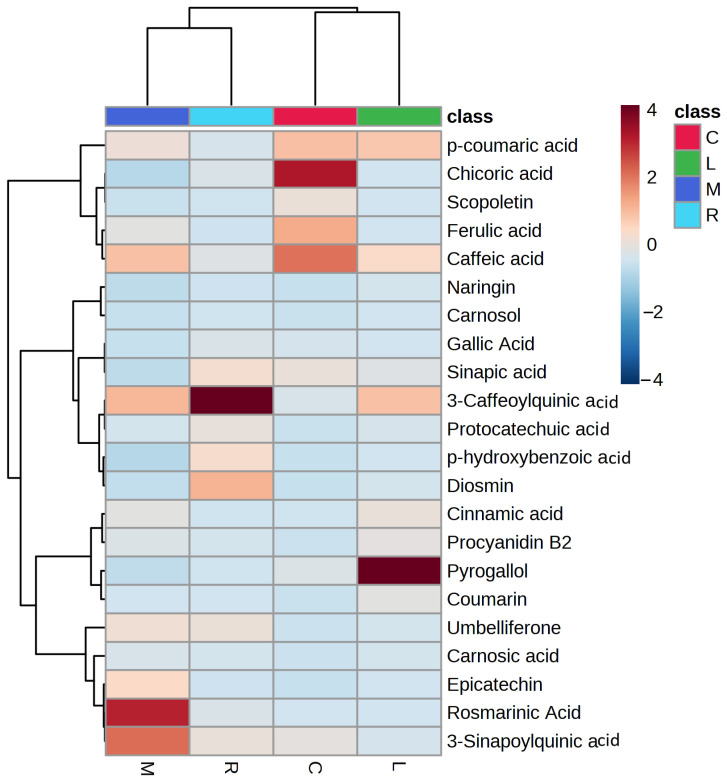
Clustered hierarchical heatmap showing quantified compounds from lemongrass (L), moringa (M), ryegrass (R), and chicory (C).

Table S1 lists the twenty-two quantified compounds that were measured in the lemongrass, moringa, ryegrass, and chicory plants. The most common type of compound in these plants is flavonoids. Moringa had the highest concentration of hydroxycinnamic acids, with rosmarinic acid having the highest concentration (748.32 ± 25.82 μg/g) 3-caffeoylquinic acid (103.93 ± 12.51 μg/g), 3-sinapoylquinic acid (125.23 ± 19.42 μg/g), and caffeine (264.05 ± 11.54 μg/g). Cinnamic acid was present in only three samples, lemongrass, chicory, and moringa (91.38 ± 8.87, 17.71 ± 1.18, and 31.32 ± 5.06, respectively). The lowest chlorogenic acid content was measured in moringa, whereas chlorogenic acid (3-caffeoylquinic acid) is the most prevalent phenolic acid in ryegrass (445.62± 31.52 μg/g). It was previously measured [36] to determine the amount of chlorogenic acid in nonconventional plants. Only chicory (286.12 ± 31.27 μg/g) and ryegrass (15.82 ± 1.59 μg/g) contain chicoric acid; *p*-hydroxybenzoic acid was measured in lemongrass (36.13 ± 0.73 g/g) and ryegrass (75.92 ± 5.67 μg/g). Lemongrass had the highest content of caffeic acid (366.78 ± 14.77 μg/g), and chicory plant extract had the lowest concentration (245.67 ± 21.55 g/g). Ryegrass plants also contained protocatechuic acid (63.14 ± 3.58 μg/g) and sinapic acid (51.05 ± 5.12 μg/g). Chicory contained gallic acid (23.59 ± 2.04 μg/g) and p-coumaric acid (154.90 ± 12.62 μg/g). Additionally, procyanidin B2 was detected to be highest in moringa (68.76 ± 4.12 μg/g) and lowest in lemongrass (44.62 ± 6.32 μg/g). Ryegrass had the highest content of diosmin (132.63 ± 11.06 μg/g), whereas moringa had the lowest value (9.51 ± 1.43 μg/g). Additionally, MetaboAnalyst 5.0 (www.metaboanalyst.ca accessed on 22 March 2023) was used to perform hierarchical heatmap clustering (Figure 3).

The heatmap figure shows that chicory included higher quantities of chicoric acid, ferulic acid, and caffeic acid than other measured phenolic components; moringa had higher quantities of rosmarinic acid, 3-sinapoylquinic acid, umbelliferone, carnosic acid, coumaric, procyanidin B2, and epicatechin. Ryegrass was found to have the highest levels of chlorogenic acid (3-caffeoylquinic acid), sinapic acid, protocatechuic acid, p-hydroxybenzoic acid, and diosmin; p-coumaric acid, caffeic acid, and pyrogallol were all present in higher concentrations in lemongrass.

### 2.8. In Silico Molecular Docking of Phenolic Compounds

**Figure 4:**
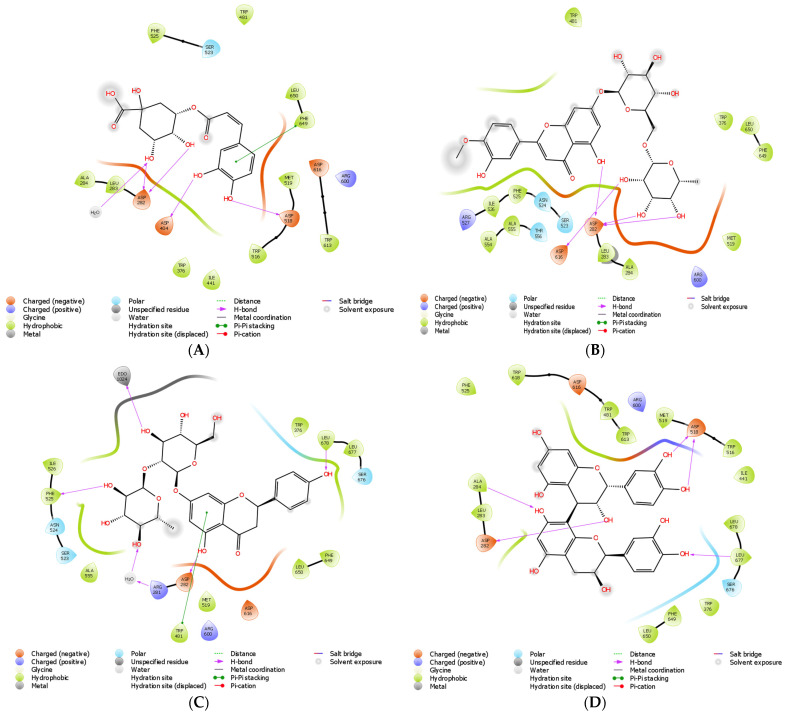
Two-dimensional binding geometry of chlorogenic acid (**A**), diosmin (**B**), naringin (**C**), and procyanidin B2 (**D**).

By using in silico molecular docking, the roles of several phenolic compounds in α-glucosidase inhibitory activities were anticipated. By employing computational methods, it is feasible to predict with high accuracy the affinities and modalities of attachment of a target molecule (or ligand) to a particular protein (or receptor), for instance, molecular docking. The projected two-dimensional binding geometries of chlorogenic acid (A), diosmin (B), naringin (C), and procyanidin B2 (D) in the α-glucosidase protein (5NN8), in addition to the computed binding energies, are shown in Figure 4 and Table S2. Chlorogenic acid (Figure 4A) formed one hydrogen bond to each of the ASPs 404 and 518, an additional bond with a molecule of water, two with ASP 282, and one with the hydrophobic PHE 649, a process known as “π–π staking” with the same substance. Diosmin (Figure 4B) established five hydrogen bonds with the negatively charged ASP 282 and one with the analogously charged ASP 616. As part of its hydrogen bonding interactions, naringin (Figure 4C) formed interactions with PHE 525, EDO 1024, ASP 282, ARG 281, and LEU 678, along with one π–π stacking contact with TRP 481. Procyanidin B2 (Figure 4D) in 7E3I interacted with PHE 295 and TRP 86, and formed hydrogen bonds with THR 83, ASP 74, TYR 124, HID 447, and ASN 87.

The protein structure of 5NN8 was docked to punicafolin. Punicafolin was positioned in the binding region of 5NN8 throughout the molecular docking procedure and enabled interaction with the nearby amino acid residues. PHE 649 is reported to be a hydrophobic amino acid. Additionally, punicafolin formed hydrophobic connections with residues of the amino acids ALA 284, PHE 525 along with TRP 481. Additionally, quercitrin and myricitrin formed two hydrogen bonds with the negatively charged ASP 282 along with one each to the negatively charged ASP 616, the hydrophobic ALA 284, and the electronegative EDO 1024. They possessed a single π–π binding with TRP 481 and a single π–π stacking hydrophobically with PHE 525. Acarbose formed twelve hydrogen bonds, two with ASPs 518, 404, 282, and 523, and three OH groups from water molecules, which also formed hydrogen bonds with ASPs 645 and 281. Rutin specifically created four hydrogen bonds, including ones with ASP 404, 616, and 518.

The binding energies of punicafolin, rutin, acarbose, procyanidin B2, myricitrin, 3-feruloylquinic acid, taxifolin, diosmin, quercitrin, chlorogenic acid, quercetin-3-O-arabinoside, naringin, 3-p-coumaroylquinic acid, myricetin, quercetin, Isorhamnetin, quinic acid, luteolin, (-)-epicatechin, hesperetin, gallic acid, 3-O-sinapoylquinic acid, diosmetin, naringenin, p-hydroxybenzoic acid, salicylic acid, caffeic acid, p-coumaric acid, pyrogallol, chrysin, protocatechuic acid, 3-4-5-trimethoxyflavone, cinnamic acid, and coumarin in 5NN8 were calculated as −12.16, −11.14, −11.05, −10.95, −10.59, −10.32, −10.13, −9.84, −9.72, −9.62, −9.49, −9.40, −9.35, −9.28, −6.95, −6.68, −6.65, −6.52, −5.36, −6.28, −6.14, −5.91, −5.36, −5.33, −5.30, −5.20, −5.08, −4.80, −4.74, −4.19, −4.12, −4.09, −4.04, and −3.93, respectively. Punicafolin is expected from the given table to have a higher binding affinity than the other chosen phenolic compounds. Punicafolin, rutin, and acarbose (standard) have stronger ability of α-glucosidase inhibition than other compounds. It is interesting to note that myricitrin has a greater affinity for binding than 3-feruloylquinic acid, taxifolin, diosmin, quercitrin, chlorogenic acid, quercetin-3-*O*-arabinoside, naringin, 3-*p*-coumaroylquinic acid, or myricetin. Comparatively, Isorhamnetin, quinic acid, luteolin, (-)-epicatechin, hesperetin, and gallic acid have lower binding affinities than myricetin. In silico molecular docking is a prediction of possible interactions between target proteins (5NN8) and potential inhibitors. Therefore, it is critical to assess the inhibitory activities of individual purified phenolic compounds to establish the precise roles of individual bioactive compounds in the inhibition of α-glucosidase. Moreover, the insights into inhibitory mechanisms of bioactive polyphenolic compounds against α-glucosidase and other proteins involved in diabetic conditions can be revealed through advanced molecular dynamics techniques and free-energy calculations, and through inverse molecular docking.

### 2.9. ADMET Properties of Abundant Phenolic Compounds

Bioinformatics facilitates the effective continuation of pharmacokinetic studies by reducing the amount of experimental effort required to evaluate various metabolites [54]. This study examined the metabolites for potential bioavailability and therapeutic effects using pharmacokinetics characteristics such as absorption, distribution, metabolism, excretion, and toxicity (ADMET) screening. The major cause of drug molecules failing in clinical tests is that they take too long to produce results are unfavorable properties of ADMET in the biological system [55]. This study examined the ADMET characteristics of the most prominent phenolic compounds found in given plant extracts.

#### 2.9.1. Absorption and Distribution

**Figure 5:**
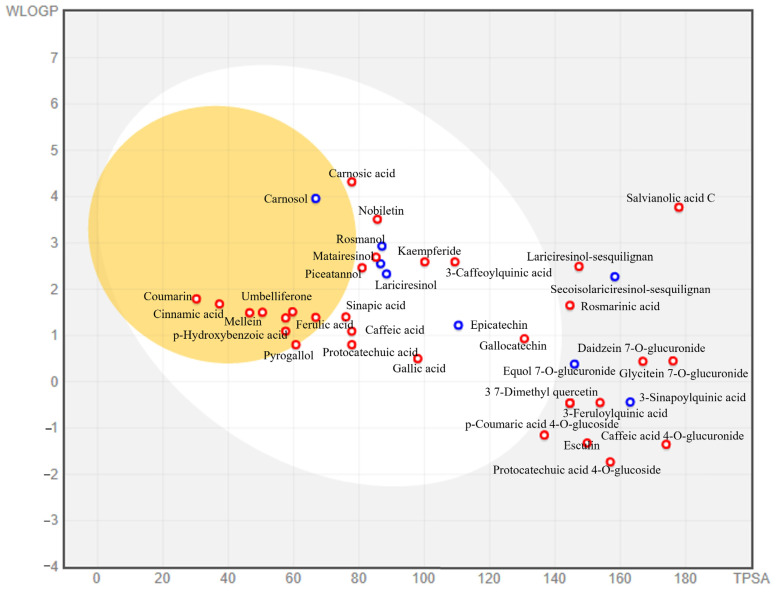
The BOILED-Egg method for monitoring phenolic chemicals, which are widely present. The red dots denote molecules that are not expected to be expelled by P-glycoprotein from the CNS, whereas the blue dots denote compounds expected to be expelled from the CNS.

With the help of the pkCSM platform and the BOILED-Egg approach (Figure 5), the absorption of the phenolic compounds had been expected. Tables S3 and S4 and Figure 5 display the absorption data. It was expected that fourteen compounds, including carnosic acid, nobiletin, sinapic acid, caffeic acid, and others, would be absorbed in the digestive system, whereas ten compounds would be expected to pass through brain barriers. Cinnamic acid, which is present in moringa, lemongrass, and chicory, was also predicted to penetrate the blood–brain barrier more rapidly than other phenolic substances (Table S4).

Nineteen substances have an absorption rate of more than 80% in the human digestive tract, based on absorption prediction including p-Hydroxybenzoic acid (83.9%), p-coumaric acid (93.5%), cinnamic acid (94.8%), coumarin (97.3%), ellagic acid (86.7%), and a few others. Finding shown in Table S4 indicate that only the coumarin molecule is projected to have high skin permeability. In addition, the highest Caco-2 cell permeability is predicted for p-Hydroxybenzoic acid (1.151), *p*-coumaric acid (1.21), resveratrol (1.17), cinnamic acid (1.717), coumarin (1.649), benzoic acid (1.707), nobiletin (1.306), dihydrobiochanin A (0.981), 4-hydroxycoumarin (1.206), scopoletin (1.184), and dihydroquercetin (0.924). A chemical is said to have high Caco-2 permeability if the value is more than 0.90 [54]. Furthermore, compounds with Caco-2 permeability, intestinal uptake, a high bioavailability score, Lipinski’s rule of compliance, failure to cross the blood–brain barrier, inability to serve as P-gp substrates, and poor skin penetration should be effective medicines [56]. Most of the compounds that are not absorbed in the digestive tract can be converted into other derivatives by microbes in the gut and absorbed in the colon [57].

**Figure 6:**
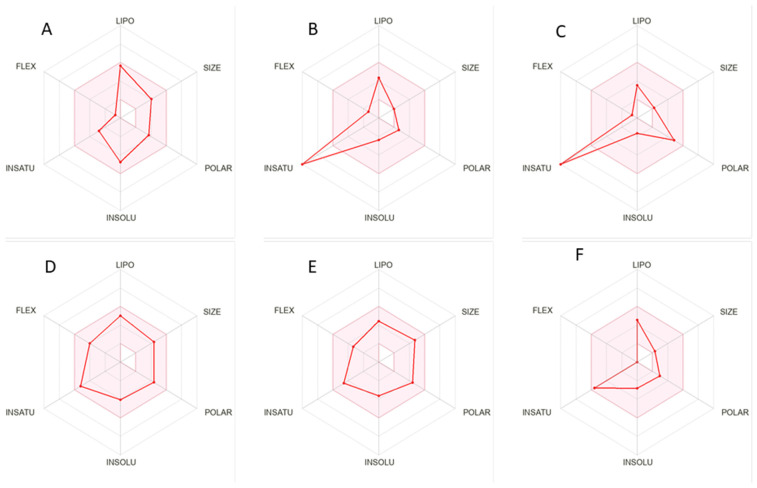
Radars to assess the oral bioavailability of Carnosol (**A**), Cinnamic acid (**B**), Gallic acid (**C**), Matairesinol (**D**), Medioresinol (**E**), and Mellein (**F**).

Flavonoids are linked to albumin after absorption and transported to the liver via the portal vein. However, flavonoids have a low bioavailability due to their restricted absorption, significant metabolism, and rapid excretion [58]. By using Diana’s approach [59], oral bioavailability of a few compounds are shown with radars (Figure 6). No substance exhibited anticipated oral bioavailability, as shown in Figure 5 and Table S5. Six physiochemical characteristics (size, polarity, lipophilicity, flexibility, saturation, and solubility) were analyzed by using the bioavailability radar to forecast the oral bioavailability of given metabolites [60].

#### 2.9.2. Metabolism, Excretion, and Toxicity

A key function of the metabolism of bioactive chemicals (drugs) is played by cytochrome P450 (CYP) [56]. Table S7 provides information on the phenolic compounds’ anticipated metabolism and excretion. The CYP model (CYP1A2, CYP2D6, CYP3A4, CYP2C9, and CYP2C19) was used to estimate metabolism for substrate or inhibitor antioxidants. Higher amounts of other bioactive compounds may come from bioactive chemicals that inhibit the CYP process, increasing their toxicity, and vice versa. It is hypothesized that bioactive substances with higher overall clearance have increased bioavailability and hepatic metabolism (Table S6). Table S7 provides a simulated toxicity screening for the bioactive chemicals. The predicted results show that none of the bioactive substances inhibits the hERG 1 channel.

## 3. Materials and Methods

### 3.1. Materials

In all experiments, only analytical-grade chemicals were used. Sigma Aldrich (Darmstadt, Germany) provided the chemicals for the characterization and identification of compounds. Folin–Ciocalteu’s reagent, hydrated sodium acetate, vanillin, sodium phosphate dibasic heptahydrate, trichloroacetic acid, ethylenediaminetetraacetic acid (EDTA), ammonium molybdate, catechin, 2,2-diphenyl-1-picryl-hydrazyl-hydrate (DPPH), potassium ferrocyanide (III), 2,4,6 tripyridyl-s-triazine (TPTZ), 2,2’-azino-bis 3-ethylbenzothiazoline-6-sulfonic acid (ABTS), 3-hydrobenzoic acid, and quercetin were purchased from the Sigma Aldrich (Castle Hill, NSW, Australia) for the estimation of polyphenols and antioxidant capability.

### 3.2. Sample Preparation and Method Optimization for Extraction of Phenolic Compounds

Chicory, lemongrass, moringa, and ryegrass plants were all collected from fields of Faisalabad division, Pakistan. Samples were reconfirmed and identified from the Department of Plant Sciences, Quaid-i-Azam University, Islamabad, Pakistan. The plant samples were dried at room temperature for two days and oven-dried at 45 °C for three days. A fine powder was prepared by grinding using a laboratory grinder. The method of extraction was optimized using different solvents: 80% ethanol, 80% methanol, 80% acetone, 80% chloroform, and Milli-Q water. The following procedure was employed to extract phenolic compounds: Using a 2 g sample and 30 mL of 80% solvent (methanol, ethanol, acetone, chloroform) in Milli-Q water, extracts from the four selected edible plants were prepared in triplicate. Samples were extracted after shaking in an orbital shaker (ZWYR-240) for 16 h at 150 rpm and 4 °C. After centrifuging the samples at 8000 g for 20 min, the supernatant was separated from the solution and filtered using a 0.45 μm syringe filter. Afterward, samples were kept at −20 °C for a maximum of seven days before LC-MS/MS and spectrophotometric analysis.

### 3.3. Total Phenolic Content and Antioxidant Potential

#### 3.3.1. Determination of Total Phenolic Content

The phenolic compound profile of samples was analyzed using the previously described method by Ali et al. [61]. First, 25 µL of Folin–Ciocalteu reagent (25% *v*/*v*) was taken with 200 µL of distilled water. Then, 25 µL of sample extract was added and incubated at 27 °C for 5 min. At last, 25 µL of sodium carbonate (10% *w*/*w*) was added to the reaction mixture and placed in incubation again in the dark at 27 °C for 1 h. Absorbance of the samples was recorded at 760 nm. Quantification of the total phenolic content was carried out by making a standard curve against gallic acid that ranged from 0 to 200 µg/mL in methanol. Results were recorded using units of GAE (milligram gallic acid equivalents) per gram of sample. 

#### 3.3.2. Total Flavonoid Content

Flavonoid content in the samples was analyzed using the method described by Ali et al. [62], with some modifications. The aluminum chloride colorimetric method was used to determine the TFC. An 80 µL sample extract was taken in 96-well plates and mixed with 80 µL AlCl_3_ solution and 120 µL sodium acetate aqueous solution (50%). After preparation of the reaction mixture, the sample was incubated in the dark at 27 °C for 2.5 h and absorbance was recorded on the spectrophotometer at 440 nm. For the quantification of flavonoid content, a standard curve (R^2^= 0.999) was constructed against 0–50 µg/mL of quercetin in methanol. The milligram quercetin equivalents per gram of the sample unit was used to express the results.

#### 3.3.3. Total Tannin Content

TTC was carried out using a modified version of the Ali et al. [62] technique. First, 150 µL vanillin solution (4%) and 25 µL of the sample solution was added. Then, 25 µL of 32% H_2_SO_4_ was then poured into the mixture. The final sample was incubated at 25 °C for 15 min. The absorbance was determined at 500 nm and the standard catechin curve (0–1000 µg/mL) was constructed. The data are given as mg CE/g.

### 3.4. Antioxidant Activities of Edible Plants

#### 3.4.1. ABTS Radical Scavenging Assay

ABTS assay was performed by following the methods described by Ali et al. [61] and Severo et al. [63]. In order to produce ABTS+ solution, a mixture of 140 mM potassium persulfate solution and 7 mM ABTS solution was incubated for 16 h in the dark. The solution was then diluted with ethanol to obtain an absorbance reading of 0.70 ± 0.02 at 734 nm. In a 96-well plate, 290 µL of ABTS+ solution and 10 µL of sample extract were added and incubated for 6 min at ambient temperature. The absorbance was measured at 734 nm. The measurement (reported as mg AAE/g) was performed by generating the standard curve against ascorbic acid values of 0–150 μg/mL in water.

#### 3.4.2. DPPH Radical Scavenging Assay

The DPPH assay for all samples was performed in triplicate by following the method described by Fia et al. [64] with modifications. First, 25 µL sample extracts were mixed with 275 µL of DPPH dye (0.1M) in methanol using the 96-well plate method. The prepared reaction mixture was then incubated for 30 min at 25 °C in a dark place and recorded at 517 nm on the spectrophotometer. The radical scavenging capability was analyzed by constructing a standard curve against ascorbic acid (0–50 µg/mL) in water. The results observed were recorded as milligrams of ascorbic acid equivalents per gram of sample (mg AAE/g). 

#### 3.4.3. Hydroxyl Radical Scavenging Assay 

The Fenton-type reaction method was used to determine the ^•^OH-RSA (hydroxyl radical scavenging activity) of the samples by following the method of Ali et al. [61]. In this method, 50 µL of anhydrous ferrous sulfate, FeSO_4_7H_2_O (6 mM) and 50 µL of 6 mM hydrogen peroxide (30%) were mixed with 50 µL sample extract and incubated for 10 min at 25 °C. Then, 50 µL of 6 mM 3-hydroxybenzoic acid was added to the solution and the absorbance was assessed at 510 nm wavelength. Ascorbic acid concentrations between 0 and 300 μg/mL were used to construct a standard curve, and results are expressed in mg AAE/g.

#### 3.4.4. Fe^2+^ Chelating Activity (FICA)

Ferrous ion chelating activity was determined by slightly modifying the methodology described by Patel [65] and Ali et al. [62]. In this method, 85 µL of water, 50 µL of ferrous chloride (2 mM), and 50 µL of ferrozine (5 mM) were mixed with 15 µL of extract and incubated at 25 °C for 10 min. The absorbance was determined at 562 nm wavelength. A standard curve was developed using EDTA at 0 to 50 μg/mL for quantification, and the data are expressed as mg EDTA/g.

### 3.5. Alpha-Glucosidase Inhibition Activity

By using the technique described by Xiong et al., alpha-glucosidase enzyme solution can be generated from intestinal acetone powder. Briefly, 25 mL of potassium phosphate buffer (0.12 M with 1% NaCl, pH 6.8) was supplemented with one gram of intestinal acetone powder. A Q55 sonicator (QSonica, CT, USA) was used to sonicate the mixture for 3 min at 50 Hz. The mixture was then centrifuged at 4000× *g* for 30 min at 4 °C. After collecting the supernatant, it was centrifuged once more for 20 min at 14,000× *g*. The experiment was finished in two days after the supernatant was kept in a freezer at −20 °C. The Bradford assay was used to determine the protein concentration of the α-glucosidase (4.99 ± 0.27 mg/mL), employing bovine serum albumin as the protein standard. The alpha-glucosidase inhibition assay was performed according to the modified approach of Ali et al. [62]. This was accomplished by combining 20 microliters of phenolic extract with 90 microliters of potassium phosphate buffering solution (0.12 M, pH 6.8) in a 96-well plate; 20 μL of alpha glucosidase solution was then added, and the mixture was incubated for 25 min at 37 °C. Following the incubation period, 20 μL of a 25 mM *p*NPG solution was incubated at 37 °C for 30 min. After dissolving the precipitates with 70 μL of dimethyl sulfoxide (DMSO), the absorbance was determined at 405 nm. The same formula used to calculate AChE inhibition activity was utilized to determine the inhibition of α-glucosidase in triplicate. Acarbose was employed as a reference substance.

### 3.6. LC-MS/MS Characterization of Phenolic Compounds

The separation and identification of phenolic metabolites from plant samples were performed using the method previously described by Ali et al. [60]. Phytochemicals were extracted and identified using the MassHunter Workstation Software (version B.06.00) from Agilent, Santa Clara, California, USA. An LC-ESI-Q-TOF-MS/MS (Accurate-Mass Q-TOF LC/MS Agilent 6520) equipped with Agilent HPLC 1200 series was used to analyze untargeted phenolic metabolites of native Australian plums, Davidson plums, quandong peaches, and muntries. The screening of the phenolic extracts was performed on a Synergi 4 μm Hydro-RP 80 LC column (250 × 4.6 mm) protected with a C18 ODS (4.0 × 2.0 mm) guard column (Phenomenex, Torrance, CA, USA). During the injection of an aliquot of 10 μL from each phenolic extract, the flow rates of mobile phase A (0.1% formic acid in Milli-Q water) along with mobile phase B (0.1% formic acid in acetonitrile) were 600 μL/min with the gradient as follows: 0–10 min (10–20% B), 10–20 min (20–25% B), 20–30 min, (25–30% B), 30–40 min (30–45% B), 40–50 min (45–60% B), 50–65 min (60–90% B), 65–67 min (90–100% B), 67–68 min (100–10% B), and 68–70 min (10% B). The auto MS/MS mode was utilized with the following LC parameters: scan mode 50–1300 amu, nitrogen gas flow rate (9 L/min) at 325 °C, capillary voltage (3500 V), nebulization 45 pressure, and collision energies (10, 20, and 40 eV). The identification and characterization of phenolic metabolites were carried out using the Agilent MassHunter Workstation Software Quality Analysis (version B.06.00) and the Personal Compounds Database Library (PCDL) for metabolites. Forty-one phenolic chemicals were semiquantified in this experiment, and each sample was run twice. In this experiment, 40 commercial standards MS/MS spectra were also obtained. Equations were produced using LC-MS/MS and a combination of 26 commercial standards [60].

### 3.7. In Silico Molecular Docking and Simulated Pharmacokinetics Study of the Most Abundant Phenolic Compounds

The pharmacokinetic properties were predicted using the pkCSM and SwissADME platforms, as described by Ali et al. [60,62].

### 3.8. Statistical Analysis

Minitab (version 18.0, Minitab, LLC, State College, PA, USA) and XLSTAT-2019.1.3 were used for analysis of variance (ANOVA), Pearson correlation, and biplot analysis. The biological activity and phenolic content data are presented as mean plus standard deviation.

## 4. Conclusions

LC-ESI-QTOF-MS/MS was used to characterize phenolic chemicals in unconventional edible plants in detail, including their identification and quantification. Our findings demonstrate that these plants are a rich source of phenolic compounds from various classes, and they further demonstrate the important part that flavonoids contribute to phenolic compounds’ aggregate. Moringa contains a higher amount of total phenolic and total flavonoids compared to other selected plants. Moringa measurements also revealed higher antioxidant and antidiabetic activity than other selected plants. Rosmarinic acid, chlorogenic acid, caffeic acid, pyrogallol, chicoric acid, and 3-sinapoylquinic acid are the most abundant phenolic compounds in the selected plants. These findings emphasize the significance of conducting thorough analyses of phenolic component profiles in natural sources, including occasionally neglected plants. Total phenolics and flavonoids in these samples showed considerable differences. Since they contain a wide variety of phenolic compounds, the complementary profile of phenolic compounds in plants makes it particularly attractive to use them as dietary supplements that enhance sensory and medicinal characteristics. Finally, these results demonstrated the antioxidant and antidiabetic activities of these plants, which can be used in many products for a variety of health-improving purposes. In silico molecular docking further helped to understand the structure–function relationship of phenolic compounds toward alpha-glucosidase inhibition activity. Researching simulated pharmacokinetics could help to understand the role of phenolic compounds in drug discovery. Additionally, this study could be helpful for researchers to screen and characterize phytochemicals in other plants.

## Figures and Tables

**Table 1:** Determination of TPC (mg GAE/g) using different solvent extractions in lemongrass, moringa chicory, and ryegrass.

Variables	Lemongrass	Chicory	Ryegrass	Moringa
80% methanol	11.91 ± 0.56 ^b^	4.76 ± 0.11 ^a^	3.43 ± 0.09 ^c^	18.0 ± 1.51 ^a^
80% ethanol	11.31 ± 0.19 ^b^	4.57 ± 0.07 ^ab^	3.12 ± 0.02 ^cd^	16.2 ± 1.33 ^b^
80% acidified methanol	12.86 ± 0.12 ^a^	4.85 ± 0.08 ^a^	3.54 ± 0.03 ^cd^	18.5 ± 1.01 ^a^
80% acidified ethanol	11.01 ± 0.41 ^b^	4.45 ± 0.14 ^ab^	3.17 ± 0.01 ^cd^	17.1 ± 1.32 ^ab^
80% acetone	8.95 ± 0.13 ^c^	3.91 ± 0.06 ^b^	1.84 ± 0.01 ^c^	16.1 ± 1.09 ^b^
80% acidified acetone	7.42 ± 0.35 ^cd^	3.76 ± 0.12 ^b^	1.63 ± 0.02 ^c^	17.2 ± 1.47 ^ab^
Water	2.91 ± 0.05 ^e^	0.45 ± 0.02 ^c^	0.24 ± 0.02 ^b^	4.01 ± 0.09 ^c^

The values (*n* = 3) are mean ± standard deviation (SD). Results with superscript letters (^a–e^) in each column are significantly different from each other.

**Table 2:** Determination of phenolic composition in plant extracts.

Samples	TPC (mg GAE/g)	TFC (mg QE/g)	TCT (mg CE/g)
Lemongrass	12.9 ± 0.12 ^b^	6.19 ± 0.09 ^b^	0.90 ± 0.76 ^a^
Chicory	4.85 ± 0.06 ^c^	1.06 ± 0.01 ^c^	2.31 ± 0.15 ^c^
Ryegrass	3.54 ± 0.08 ^c^	1.52 ± 0.13 ^c^	0.58 ± 0.05 ^c^
Moringa	18.5 ± 1.01 ^a^	10.1 ± 0.83 ^a^	1.45 ± 0.01 ^b^

TPC (total phenolic content), TFC (total flavonoid content), TCT (total condensed tannins). The values are shown as the mean standard deviation (*n* = 3) per gram of dry matter. Results in each column with superscript letters (^a–d^) are significantly different from each other (*p* ≤ 0.05). GAE (gallic acid equivalents); QE (quercetin equivalents); Catechin equivalent (CE).

**Table 3:** Antioxidant and antidiabetic potential of selected edible plants.

Variables	DPPH (mg AAE/g)	ABTS (mg AAE/g)	FICA (μg EDTA/g)	^•^OH-RSA (mg AAE/g)	α-Glucosidase Inhibition Activity (μg/mL)
Lemongrass	25.73 ± 0.18 ^a^	44.8 ± 0.93 ^a^	3.42 ± 0.11 ^a^	29.73 ± 0.48 ^a^	2.15 ± 0.13 ^a^
Chicory	16.61 ± 0.23 ^b^	35.4 ± 0.68 ^b^	2.52 ± 0.15 ^b^	17.69 ± 0.27 ^b^	16.41 ± 1.21 ^b^
Ryegrass	11.18 ± 0.21 ^c^	18.4 ± 0.56 ^c^	1.81 ± 0.05 ^c^	16.33 ± 0.39 ^b^	29.02 ± 2.17 ^c^
Moringa	34.16 ± 2.32 ^a^	54.9 ± 4.24 ^a^	7.49 ± 0.39 ^a^	41.6 ± 3.52 ^a^	1.89 ± 0.01 ^a^

The values are mean ± SD per gram dried sample in triplicate (*n* = 3). AAE (ascorbic acid equivalents); EDTA (ethylenediaminetetraacetic acid); 2,2-azio-bis-3-ethylbenzothiazoline-6-sulfonic acid assay (ABTS); (2,2-diphenyl-1-picrylhydrazyl assay (DPPH); ferrous ion chelating activity (FICA); and hydroxyl radical scavenging activity (^•^OH-RSA). Results with superscript letters (^a–c^) in each column are significantly different from each other

**Table 4:** Pearson’s correlation of biological activity and phenolic content.

Variables	TPC	TFC	TCT	DPPH	ABTS	FICA	^•^OH-RSA
TFC	**0.99**						
TCT	−0.03	−0.12					
DPPH	**0.99**	**0.97**	0.18				
ABTS	**0.93**	0.87	0.32	**0.97**			
FICA	**0.93**	**0.93**	0.15	**0.93**	0.86		
^•^OH-RSA	**0.99**	**0.99**	−0.02	**0.98**	**0.91**	**0.96**	
α-glu	**0.94**	0.89	0.11	**0.96**	**0.97**	0.79	**0.91**

Values in bold are different from 0 with a significance level of alpha = 0.1.

**Table 5:** LC-QTOF-MS/MS characterization of polyphenols from plant extracts.

No.	Retention Time	ESI +/−	Theoretical *m*/*z*	Precursor *m*/*z*	Mass Error	Product Ions	Formula	Compound Name	Samples
								Phenolic acids	
								Hydroxybenzoic acids	
**1**	6.216	[M − H]^−^	171.0288	171.0298	5.9	125	C_7_H_6_O_5_	Gallic acid	L, M, C, R
**2**	10.718	[M + H]+	155.0339	155.0341	1.3	109	C_7_H_6_O_4_	Protocatechuic acid	L, M, R, C
**3**	13.549	[M − H]−	315.0721	315.0725	1.3	153	C_13_H_16_O_9_	Protocatechuic acid 4-*O*-glucoside	L, M
**4**	15.982	[M − H]−	137.0244	137.0252	4.8	93	C_7_H_6_O_3_	*p*-Hydroxybenzoic acid	R, L, M, R
								Hydroxycinnamic acids	
**5**	6.302	* [M + H]+	399.1286	399.1279	−1.8	223, 191	C_18_H_22_O_10_	3-Sinapoylquinic acid	L, C, M
**6**	6.302	[M + H]+	311.1125	311.1133	2.6	147, 131, 103	C_15_H_18_O_7_	Cinnamoyl glucose	L, M
**7**	13.038	* [M − H]−	353.0878	353.0879	0.3	253, 190, 144	C_16_H_18_O_9_	3-Caffeoylquinic acid	L, M, C, R
**8**	13.170	[M − H]−	359.0772	359.0769	−0.8	197, 179, 161, 135	C_18_H_16_O_8_	Rosmarinic acid	C, M
**9**	13.720	[M − H]−	355.0671	355.0684	3.7	179, 161	C_15_H_16_O_10_	Caffeic acid 4-*O*-glucuronide	C, R, M
**10**	14.041	[M − H]−	385.114	385.1140	0.0	223, 193	C_17_H_22_O_10_	1-*O*-Sinapoyl-beta-D-glucose	R, L
**11**	18.203	* [M − H]−	179.035	179.0349	−0.6	143, 135, 133	C_9_H_8_O_4_	Caffeic acid	L, M, C, R
**12**	18.341	[M − H]−	367.1034	367.1035	0.3	298, 288, 192, 191	C_17_H_20_O_9_	3-Feruloylquinic acid	L, C, M, R
**13**	18.697	* [M − H]−	337.0929	337.0926	−0.9	265, 173, 162	C_16_H_18_O_8_	3-*p*-Coumaroylquinic acid	L, M
**14**	21.546	* [M − H]−	223.0612	223.0607	−2.2	193, 179, 149, 134	C_11_H_12_O_5_	Sinapic acid	L, M, R
**15**	22.719	[M − H]−	221.0455	221.0453	−0.9	163	C_11_H_10_O_5_	p-Coumaroyl glycolic acid	C, M
**16**	23.085	[M − H]−	193.0506	193.0502	−2.1	178, 149, 134	C_10_H_10_O_4_	Ferulic acid	L, M, C, R
**17**	24.020	[M − H]−	693.2036	693.2016	−2.9	193, 134	C_32_H_38_O_17_	1,2-Diferuloylgentiobiose	L, R
**18**	24.095	[M − H]−	325.0929	325.0936	2.2	163, 119	C_15_H_18_O_8_	*p*-Coumaric acid 4-*O*-glucoside	L, C
**19**	24.681	[M − H]−	295.0459	295.0471	4.1	115	C_13_H_12_O_8_	*p*-Coumaroyl tartaric acid	C, M
**20**	26.759	* [M − H]−	147.0451	147.0450	−0.7	129, 103	C_9_H_8_O_2_	Cinnamic acid	L, C, M, R
**21**	29.106	* [M − H]−	163.04	163.0402	1.2	119	C_9_H_8_O_3_	*p*-Coumaric acid	L, C, M
**22**	29.184	[M − H]−	529.1351	529.1354	0.6	193, 191, 179	C_26_H_26_O_12_	1-Caffeoyl-5-feruloylquinic acid	C, L
**23**	33.081	* [M − H]−	543.1508	543.1492	−2.9	193, 191, 134	C_27_H_28_O_12_	3,5-Diferuloylquinic acid	L, M
**24**	36.425	* [M + H]+	871.2655	871.2618	−4.2	676, 195, 177	C_42_H_46_O_20_	1,2,2′-Triferuloylgentiobiose	L, R, M
								Flavonoids	
								Flavanols	
**25**	4.873	[M − H]−	715.1304	715.1310	0.8	565, 139	C_36_H_28_O_16_	Theaflavin 3-*O*-gallate	L, R
**26**	17.002	[M − H]−	305.0667	305.0673	2.0	269, 219	C_15_H_14_O_7_	(+)-Gallocatechin	C, M
**27**	21.174	* [M − H]−	577.1351	577.1354	2.3	451, 425, 289	C_30_H_26_O_12_	Procyanidin B2	L, M, C, R
**28**	21.246	* [M + H]+	291.0863	291.0850	−4.5	291	C_15_H_14_O_6_	(-)-Epicatechin	L, M, C, R
**29**	22.814	[M + H]+	483.1133	483.1133	0.0	483	C_21_H_22_O_13_	(−)-Epigallocatechin 7-*O*-glucuronide	L, C
**30**	23.438	[M − H]−	865.1985	865.2009	4.6	739, 695, 577, 451	C_45_H_38_O_18_	Procyanidin trimer C1	C, M
**31**	23.590	[M − H]−	563.1195	563.1206	2.0		C_29_H_24_O_12_	Theaflavin	C, R
**32**	24.681	[M − H]−	457.0776	457.0780	0.9	305, 169	C_22_H_18_O_11_	(−)-Epigallocatechin 3-*O*-gallate	C, M
								Flavanones	
**33**	4.136	* [M − H]−	609.1825	609.1855	4.9	301	C_28_H_34_O_15_	Hesperidin	L, C, M
**34**	14.151	[M − H]−	595.1668	595.1659	−1.5	459, 287, 151	C_27_H_32_O_15_	Neoeriocitrin	C, R, M
**35**	20.214	* [M − H]−	579.1719	579.1739	3.5	271	C_27_H_32_O_14_	Naringin	L, M
**36**	26.216	[M + H]+	435.1286	435.1290	0.9	273	C_21_H_22_O_10_	Naringenin 7-*O*-glucoside	L, C, M
								Flavones	
**37**	11.827	* [M + H]+	403.1388	403.1394	1.5	237, 188, 145, 59	C_21_H_22_O_8_	Nobiletin	L, R
**38**	15.587	[M − H]−	577.1563	577.1583	4.2	431, 269	C_27_H_30_O_14_	Rhoifolin	C, M
**39**	19.524	[M − H]−	621.1097	621.1073	−3.9	271	C_27_H_26_O_17_	Apigenin 7-*O*-diglucuronide	R
**40**	20.550	[M − H]−	343.0823	343.0819	−1.2	327, 255, 241	C_18_H_16_O_7_	Cirsilineol	L, C
**41**	21.076	* [M − H]−	593.1512	593.1504	−1.3	449, 283	C_27_H_30_O_15_	Apigenin 6,8-di-C-glucoside	L, C
**42**	21.174	* [M − H]−	431.0983	431.1002	4.4	269	C_21_H_20_O_10_	Apigenin 6-C-glucoside	M
**43**	24.378	* [M − H]−	607.1668	607.1651	−2.8	301, 300	C_28_H_32_O_15_	Diosmin	L, M
**44**	24.681	[M − H]−	637.1046	637.1075	4.6	285	C_27_H_26_O_18_	Luteolin 7-*O*-diglucuronide	C, L
**45**	26.169	[M − H]−	461.1089	461.1094	1.1	299	C_22_H_22_O_11_	Chrysoeriol 7-*O*-glucoside	C
**46**	28.604	* [M + H]+	287.055	287.0555	1.7	287	C_15_H_10_O_6_	3,4′,7-Tetrahydroxyflavone	L, M
								Flavonols	
**47**	4.706	[M − H]−	609.1097	609.1100	0.5	301	C_26_H_26_O_17_	Quercetin 3-*O*-xylosyl-glucuronide	R
**48**	13.680	[M − H]−	627.1567	627.1570	0.5	303	C_27_H_32_O_17_	Taxifolin 4′,7-diglucoside	C, R
**49**	17.320	* [M − H]−	579.1355	579.1350	−0.9	285	C_26_H_28_O_15_	Kaempferol 3-*O*-xylosyl-glucoside	L
**50**	21.683	[M − H]−	463.0882	463.0872	−2.2	317	C_21_H_20_O_12_	Myricetin 3-*O*-rhamnoside	L, M
**51**	21.694	* [M − H]−	461.0725	461.0746	4.6	285, 113, 85	C_21_H_18_O_12_	Kaempferol 3-*O*-glucuronide	R, L
**52**	22.899	[M − H]−	298.0483	298.0475	−2.7	283, 151	C_16_H_11_O_6_	Kaempferide	L
**53**	23.391	[M − H]−	535.1093	535.1117	4.5	359	C_24_H_24_O_14_	Jaceidin 4′-*O*-glucuronide	R, C
**54**	23.701	[M − H]−	461.1089	461.1076	−2.8	315	C_22_H_22_O_11_	Isorhamnetin 3-*O*-rutinoside	L
**55**	30.894	* [M − H]−	329.0667	329.0680	4.0	314, 299, 271	C_17_H_14_O_7_	3,7-Dimethylquercetin	L, M
								Isoflavonoids	
**56**	4.501	[M + H]+	533.129	533.1297	1.3	533	C_25_H_24_O_13_	6″-*O*-Malonylglycitin	L, M
**57**	22.837	* [M − H]−	429.0827	429.0828	0.2	253	C_21_H_18_O_10_	Daidzein 7-*O*-glucuronide	L, C
**58**	23.010	[M − H]−	517.0987	517.1013	5.0	271	C_24_H_22_O_13_	6″-*O*-Malonylgenistin	M, C
**59**	26.707	[M + H]+	271.0965	271.0968	1.1	253, 137	C_16_H_14_O_4_	Dihydroformononetin	L, M
**60**	54.991	* [M − H]−	459.0933	459.0916	−3.7	441, 283, 267	C_22_H_20_O_11_	Glycitein 7-*O*-glucuronide	M, L
**61**	55.134	[M − H]−	417.1191	417.1184	−1.7	241	C_21_H_22_O_9_	Equol 7-*O*-glucuronide	M
								Stilbenes	
**62**	6.905	[M − H]−	243.0663	243.0671	3.3	225, 201, 174, 159	C_14_H_12_O_4_	Piceatannol	M, C
**63**	9.737	[M + H]+	303.1227	303.1225	−0.7	285	C_17_H_18_O_5_	3′-Hydroxy-3,4,5,4′-tetramethoxystilbene	L, M
								Lignans	
**64**	14.203	[M + H]+	389.1595	389.1595	0.0	389	C_21_H_24_O_7_	Medioresinol	L, M
**65**	18.156	* [M − H]−	555.2235	555.2220	−2.7	359	C_30_H_36_O_10_	Lariciresinol-sesquilignan	L, C, M
**66**	19.885	[M − H]−	557.2392	557.2390	−0.4	539, 521, 509, 361	C_30_H_38_O_10_	Secoisolariciresinol-sesquilignan	M
**67**	43.624	[M − H]−	359.15	359.1504	1.1	329, 192, 178, 175, 160	C_20_H_24_O_6_	Lariciresinol	M
**68**	45.726	[M − H]−	357.1343	357.1345	0.6		C_20_H_22_O_6_	Matairesinol	M
								Other compounds	
**69**	4.551	[M − H]−	345.1707	345.1693	−3.9	301	C_20_H_26_O_5_	Rosmanol	M
**70**	38.744	* [M + H]+	151.1118	151.1121	2.0	107	C_10_H_14_O	Carvacrol	L, M
**71**	45.817	[M − H]−	331.1915	331.1920	1.5	287	C_20_H_28_O_4_	Carnosic acid	L, R, M
**72**	50.044	[M − H]−	329.1758	329.1766	2.4	285	C_20_H_26_O_4_	Carnosol	L, M
**73**	62.904	[M − H]−	343.1551	343.1556	1.5	299	C_20_H_24_O_5_	Rosmadial	M
**74**	49.606	[M − H]−	315.1085	315.1097	3.8	153, 123	C_14_H_20_O_8_	Hydroxytyrosol 4-*O*-glucoside	M
**75**	4.009	* [M + H]+	127.039	127.0387	−2.4	127	C_6_H_6_O_3_	Pyrogallol	L, C, M
**76**	16.729	[M − H]−	717.1461	717.1479	2.5	520, 357, 179, 161	C_36_H_30_O_16_	Salvianolic acid B	R, C
**77**	17.201	[M − H]−	491.0983	491.0978	−1.0	311, 267, 249	C_26_H_20_O_10_	Salvianolic acid C	C, L
**78**	4.331	* [M − H]−	245.0455	245.0444	−4.5	215, 201	C_13_H_10_O_5_	Isopimpinellin	R, C, L
**79**	18.203	* [M − H]−	135.0451	135.0450	−0.7	107, 93, 79	C_8_H_8_O_2_	*p*-Anisaldehyde	L, M
**80**	14.188	[M − H]−	191.035	191.0343	−3.7	175, 147	C_10_H_8_O_4_	Scopoletin	M, C
**81**	20.611	* [M − H]−	145.0295	145.0293	−1.4	101	C_9_H_6_O_2_	Coumarin	M, L
**82**	26.806	[M − H]−	339.0721	339.0705	−4.7	177	C_15_H_16_O_9_	Esculin	M, L
**83**	26.949	* [M − H]−	161.0244	161.0247	1.9	133	C_9_H_6_O_3_	Umbelliferone	L, M
**84**	42.621	[M − H]−	177.0557	177.0553	−2.3	133	C_10_H_10_O_3_	Mellein	M, L
**85**	30.115	[M − H]−	473.0725	473.0736	2.3	293, 311	C_22_H_18_O_12_	Chicoric acid	R, C

Notes: chicory (C), ryegrass (R), moringa (M), lemongrass (L); * asterisk indicates that compound identified in both modes.

## Data Availability

The supporting data are available in the Supplementary Materials.

## Supplementary Materials

The following supporting information can be downloaded at: https://www.mdpi.com/article/10.3390/molecules28186703/s1, Figure S1: Base Peak chromatograms (BPC) of ryegrass, lemongrass, chicory and moringa in positive and negative modes; Figure S2: MS/MS spectra of some elected compounds in some selected plants; Table S1: Quantification of abundant phenolic compounds (μg/g); Table S2: The calculated binding energies of phenolic compounds; Table S3: Predicted absorption and distribution of selected compounds; Table S4: Pharmacokinetics properties of selected compounds; Table S5: Radar bioavailability properties of selected compounds; Table S6: Metabolism and excretion of selected compounds; Table S7: Predicted toxicity of abundant phenolic compounds.
