# Supplementary material for: Optimized Extraction of Polyphenols from Unconventional Edible Plants: LC-MS/MS Profiling of Polyphenols, Biological Functions, Molecular Docking, and Pharmacokinetics Study
Source: Molecules. 2023 Sep 19;28(18):6703. doi: 10.3390/molecules28186703 (PMC10534510; doi:10.3390/molecules28186703)
Supplement: Supplementary file 1 [file molecules-28-06703-s001.zip › molecules-2592831-SI.pdf]

Supplementary Material

Optimized Extraction of Polyphenols from Unconventional Edible Plants, LC-MS/MS Profiling of Polyphenols, Biological Functions, Molecular Docking and Pharmacokinetics Study

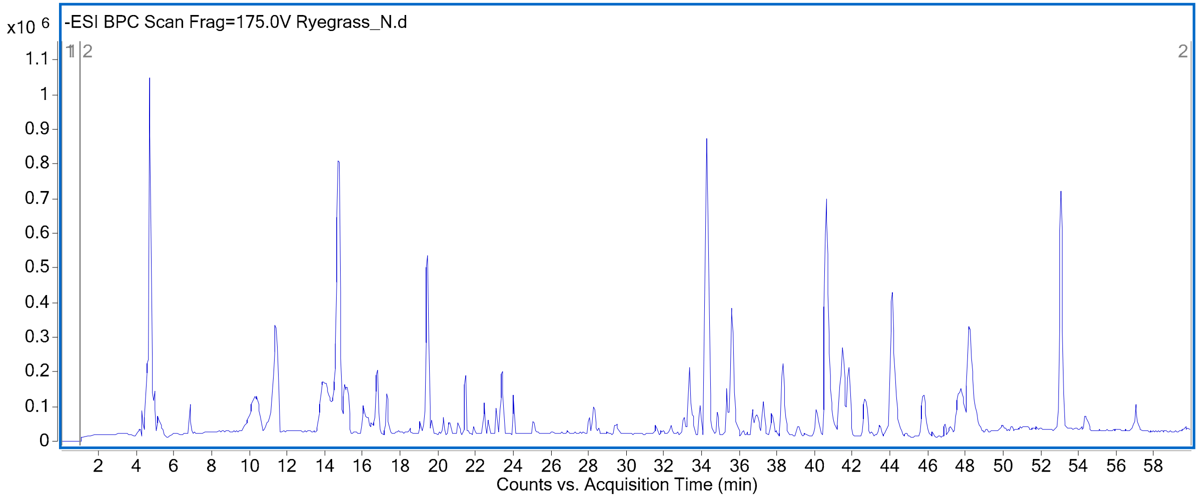

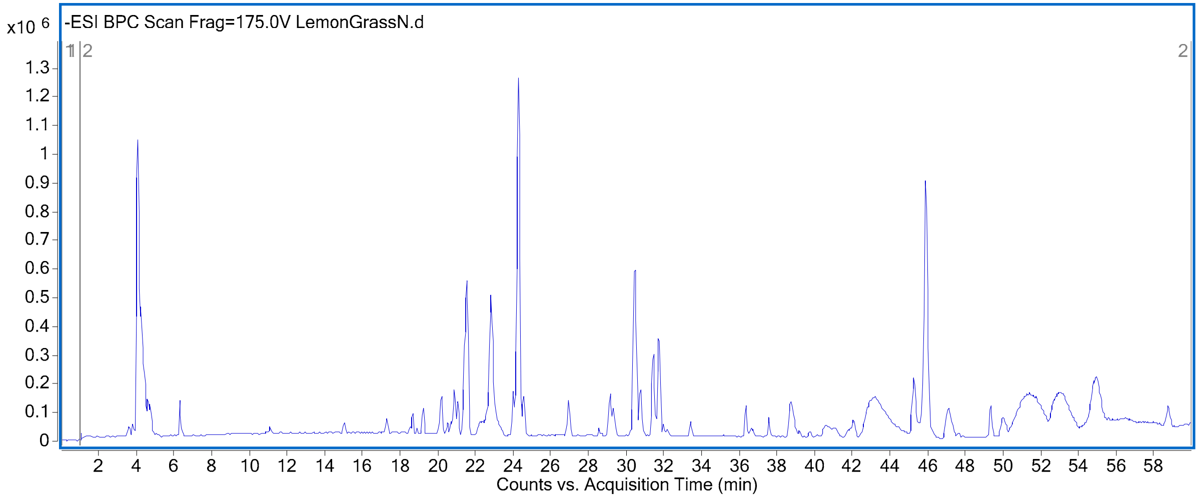

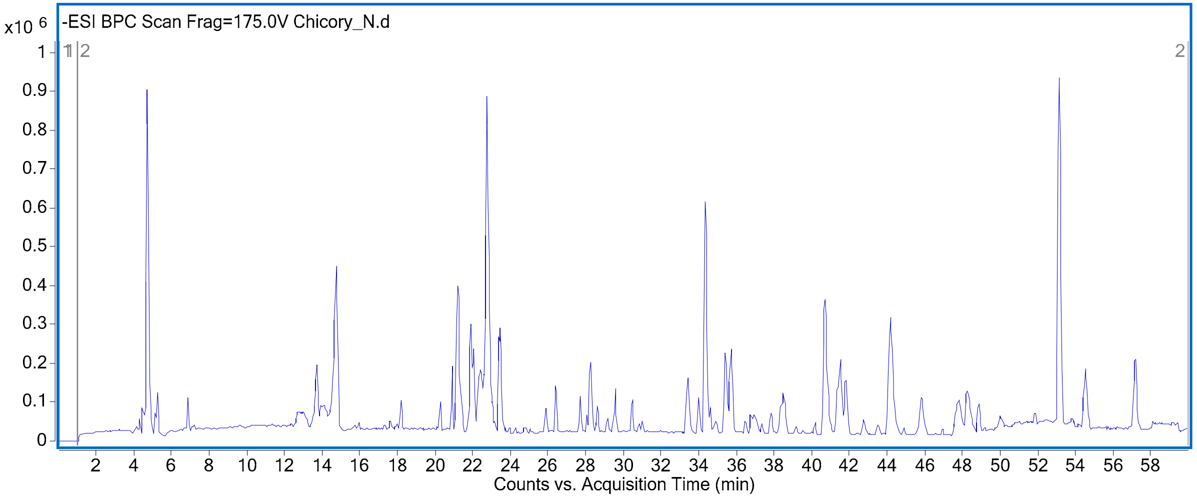
x10 6
-ESI BPC Scan Frag=175.0V Chicory_N.d
Counts vs. Acquisition Time (min)

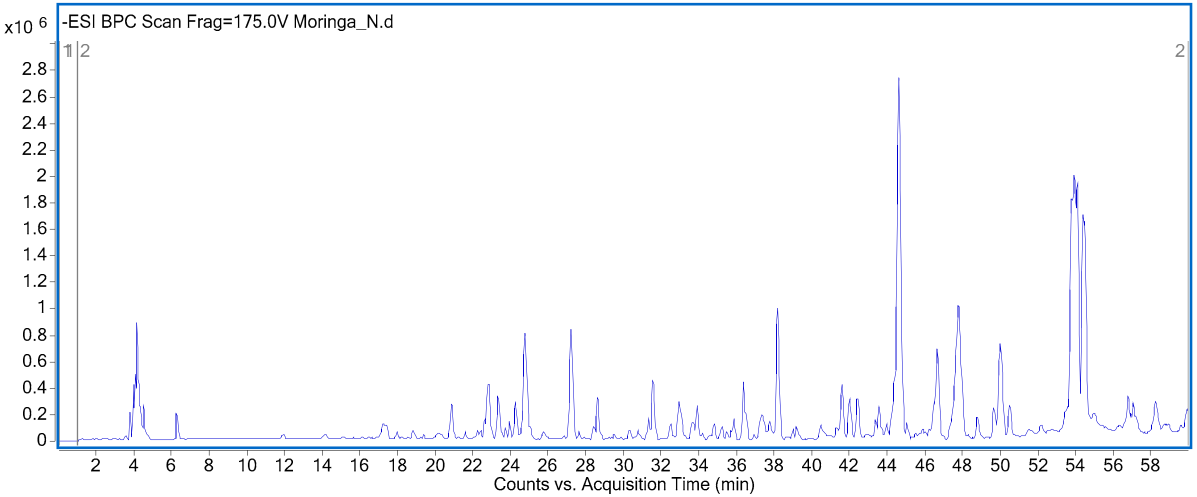
x10 6
-ESI BPC Scan Frag=175.0V Moringa_N.d
Counts vs. Acquisition Time (min)

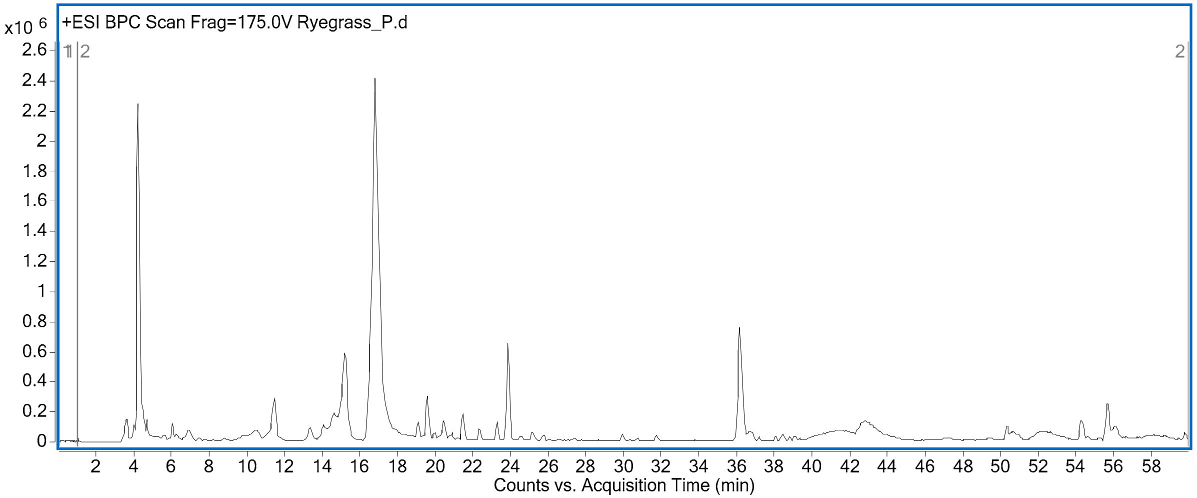
+ESI BPC Scan Frag=175.0V Ryegrass_P.d
x10 6
Counts vs. Acquisition Time (min)

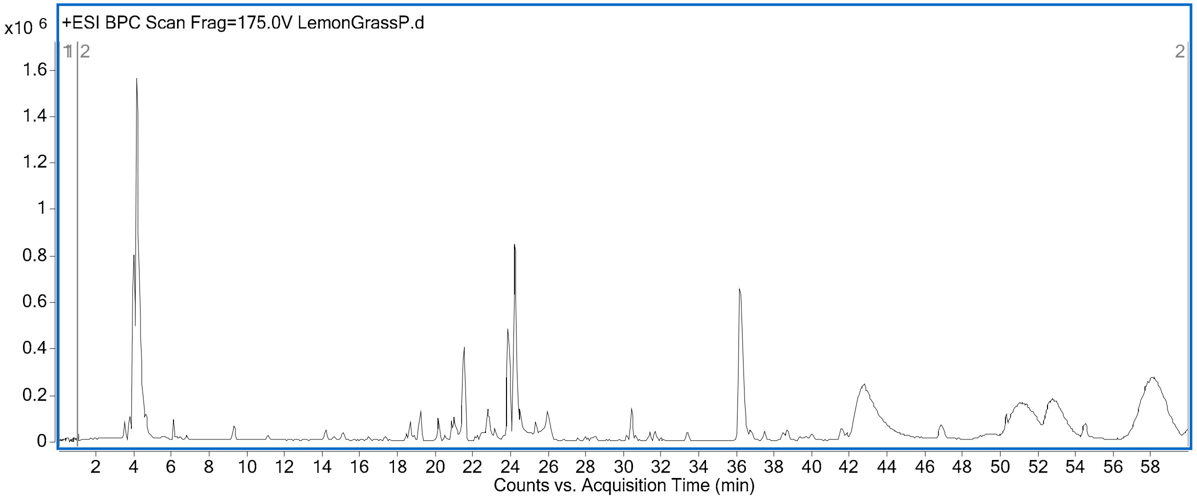
+ESI BPC Scan Frag=175.0V LemonGrassP.d
x10 6
Counts vs. Acquisition Time (min)

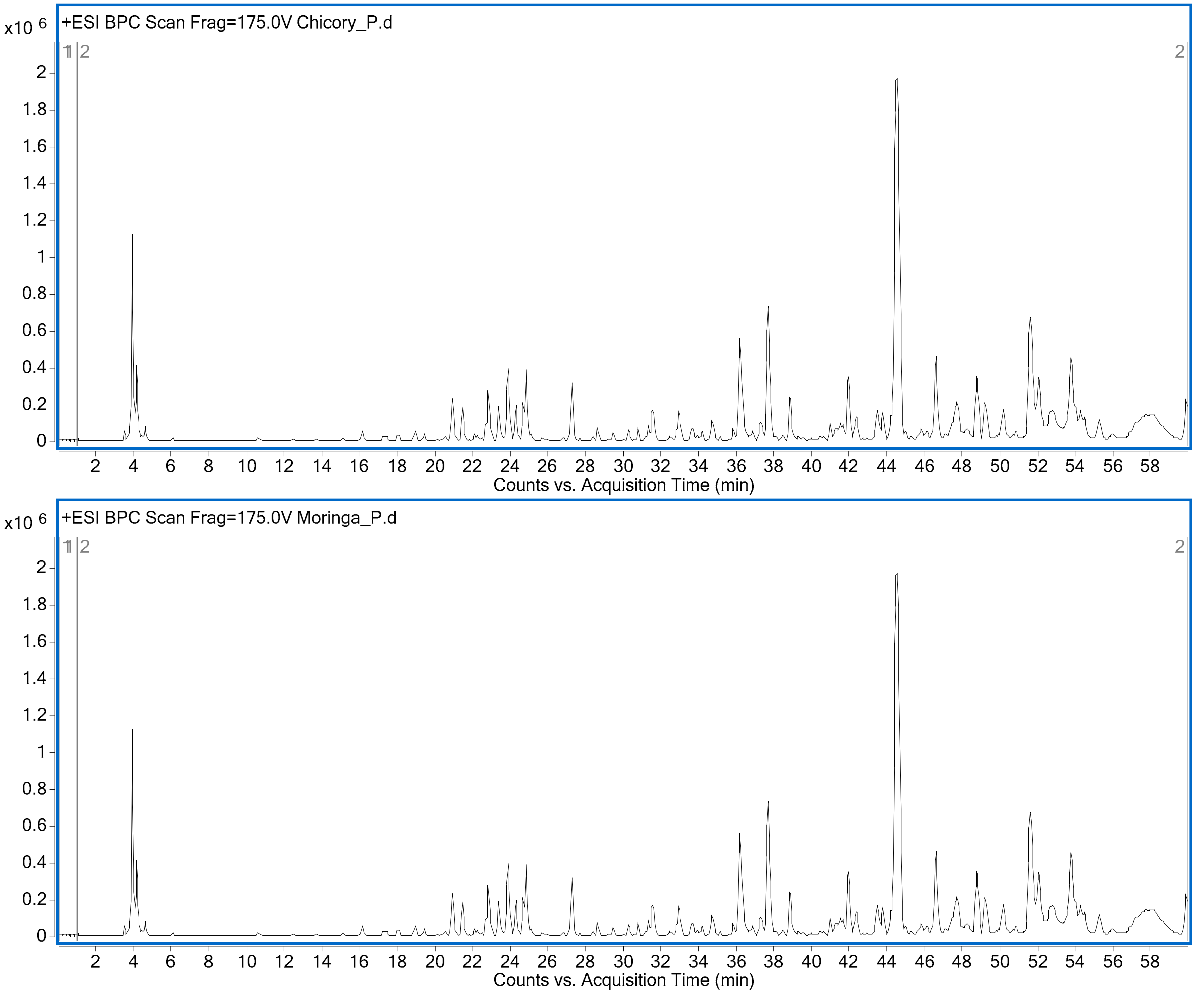

Figure S1. Base Peak chromatograms (BPC) of ryegrass, lemongrass, chicory and moringa in positive and negative modes

Chicoric acid

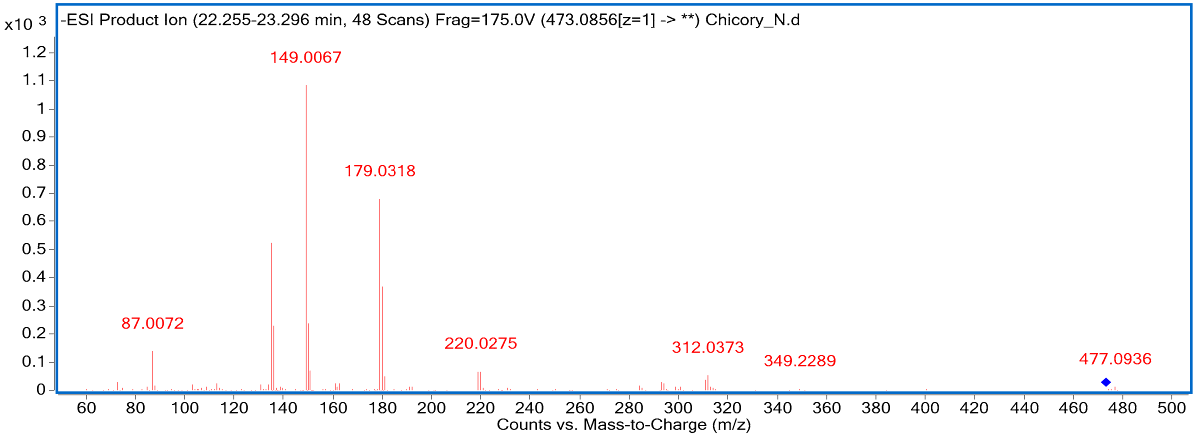

Chlorogenic acid

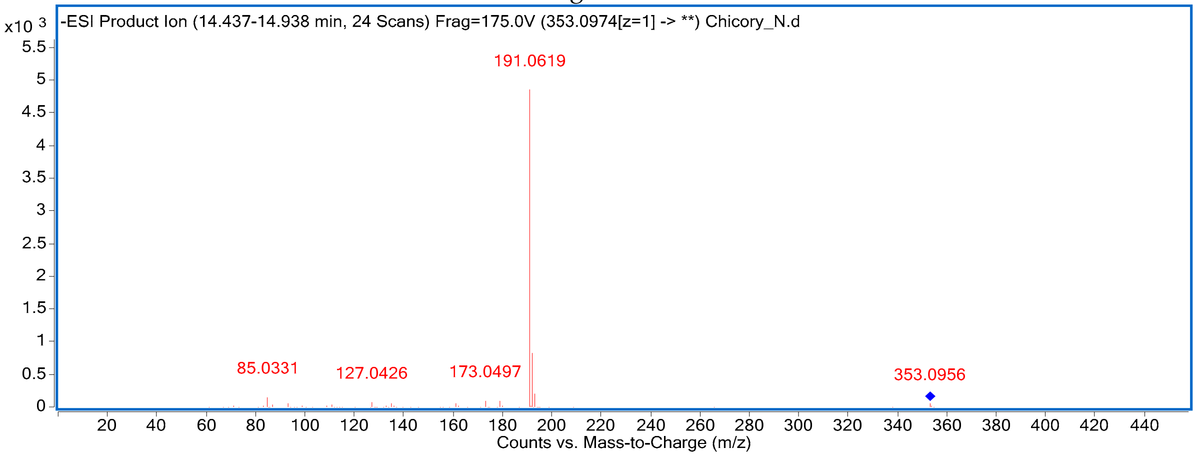

Caffeic acid

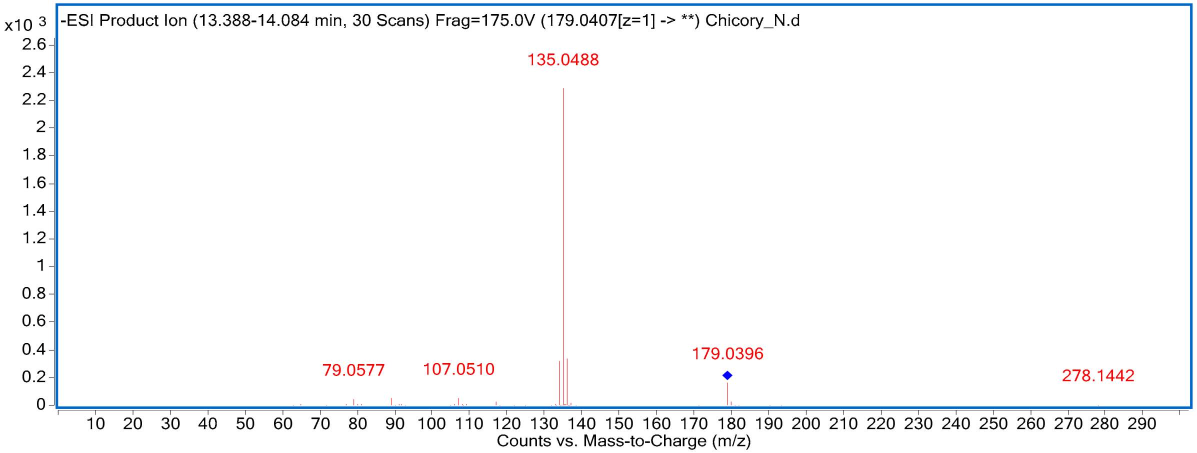

Ferulic acid

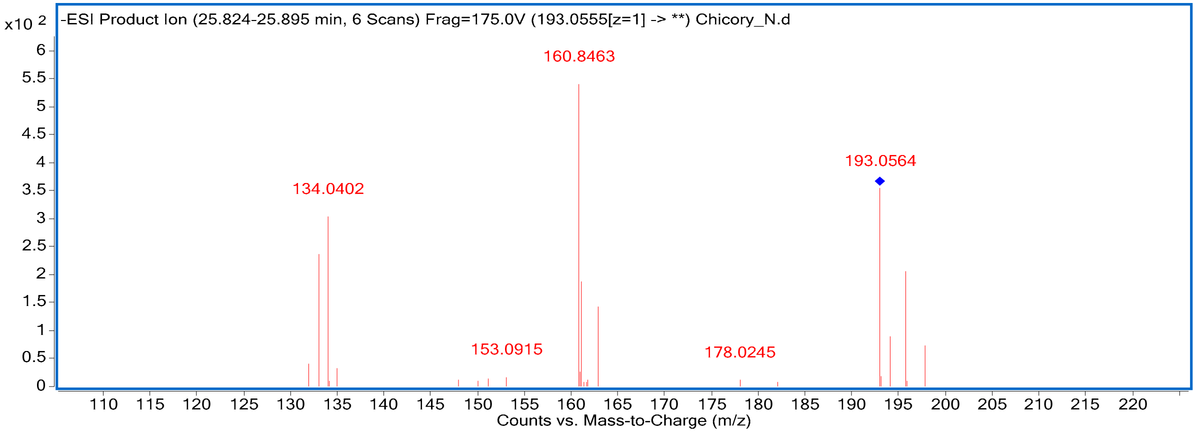

Mellein

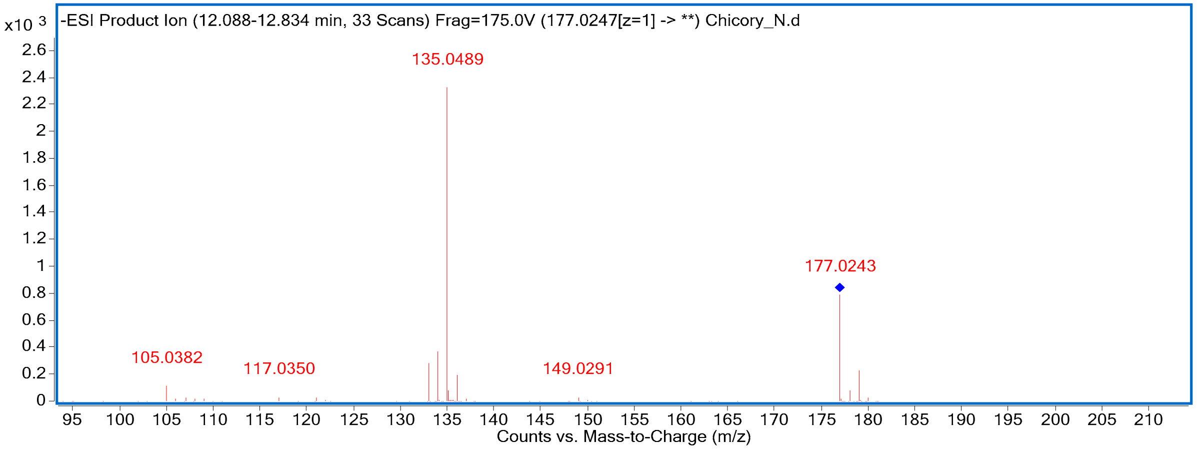

p-hydroxybenzoic acid

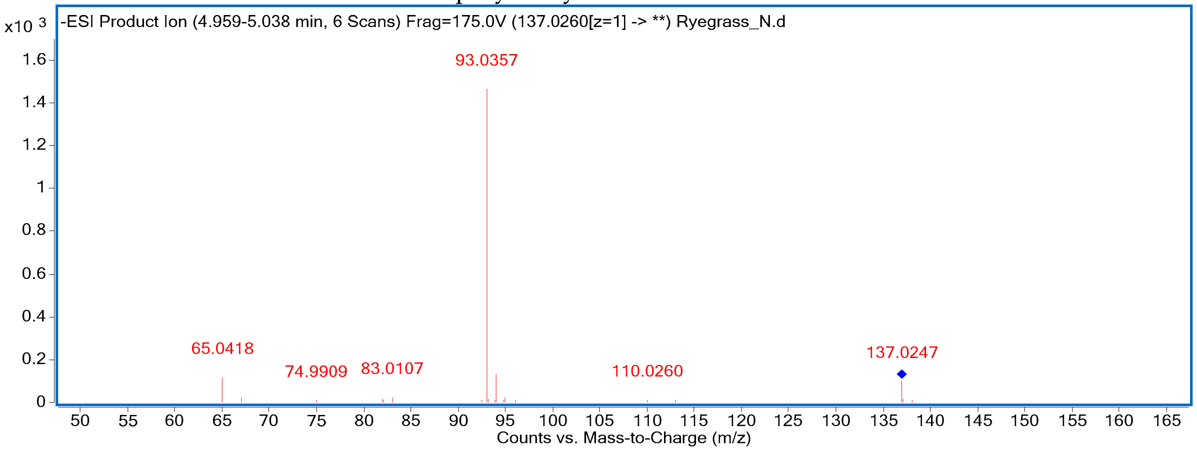

Umbelliferone

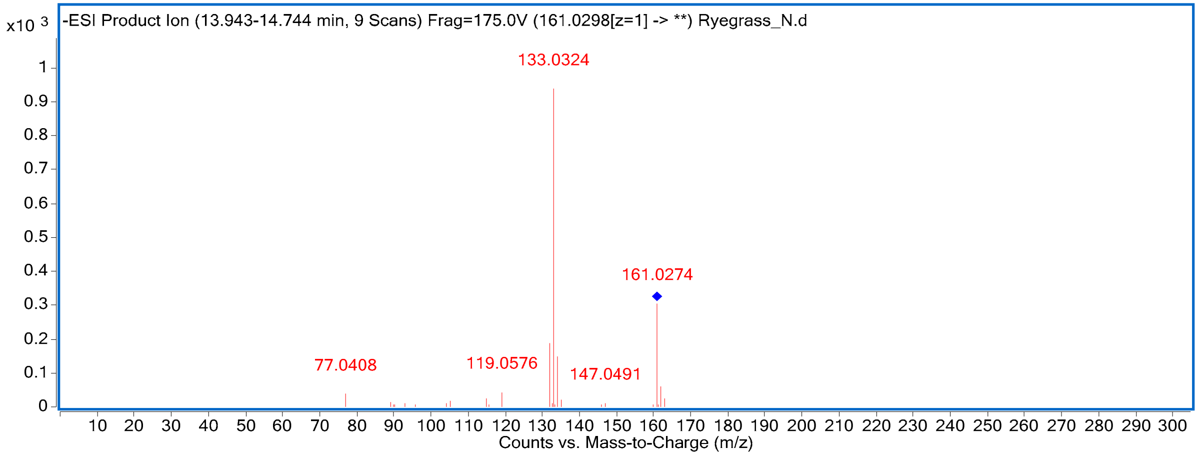

Caffeic acid

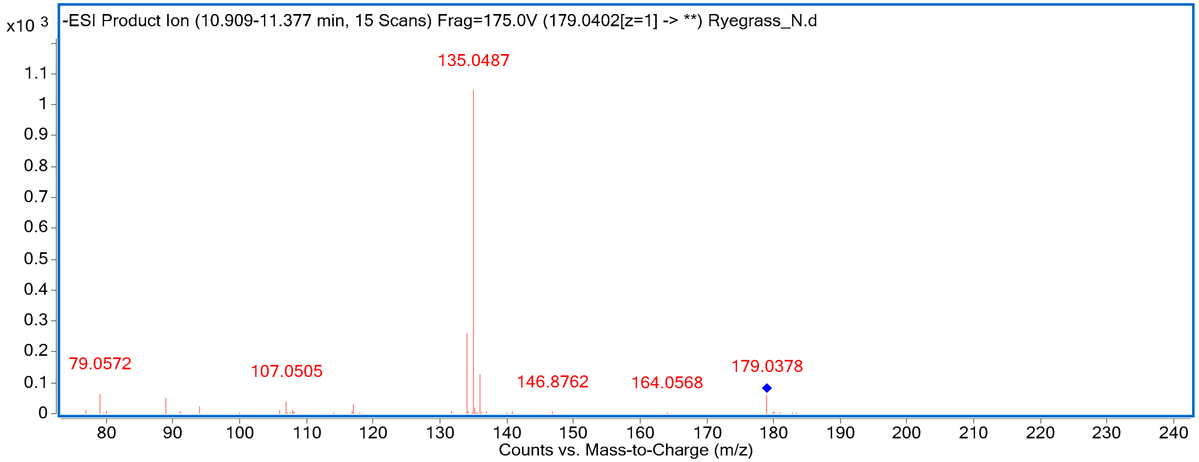

Protocatechuic acid

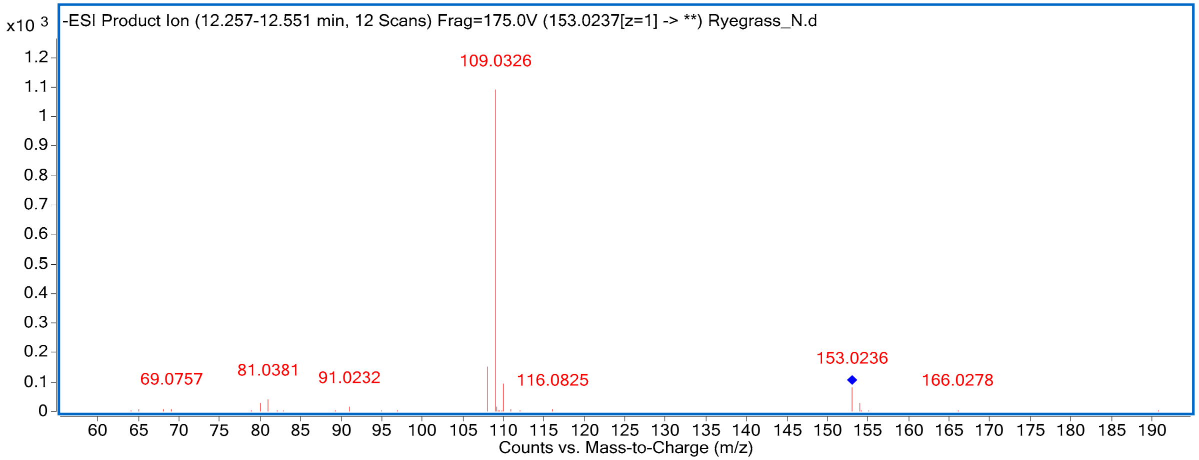

3-Feruloylquinic acid

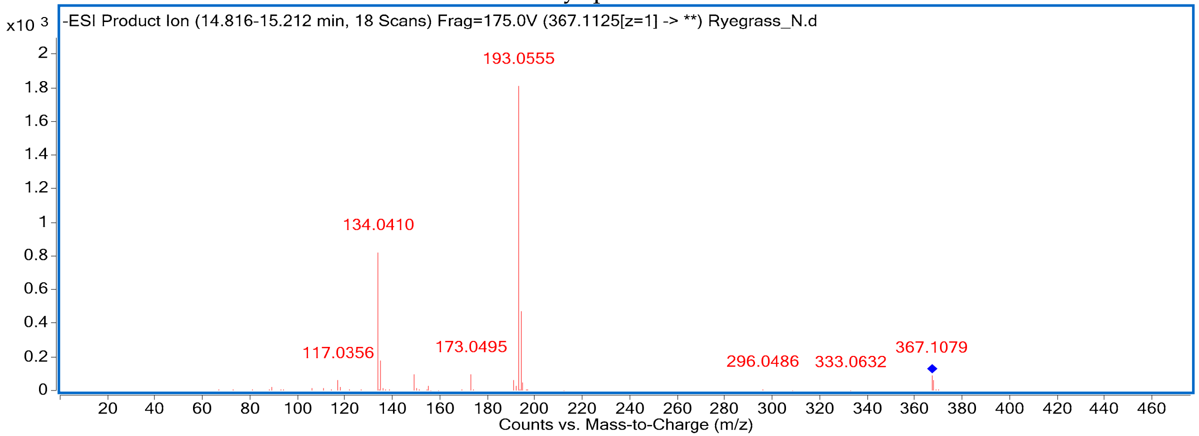

p-Coumaric acid

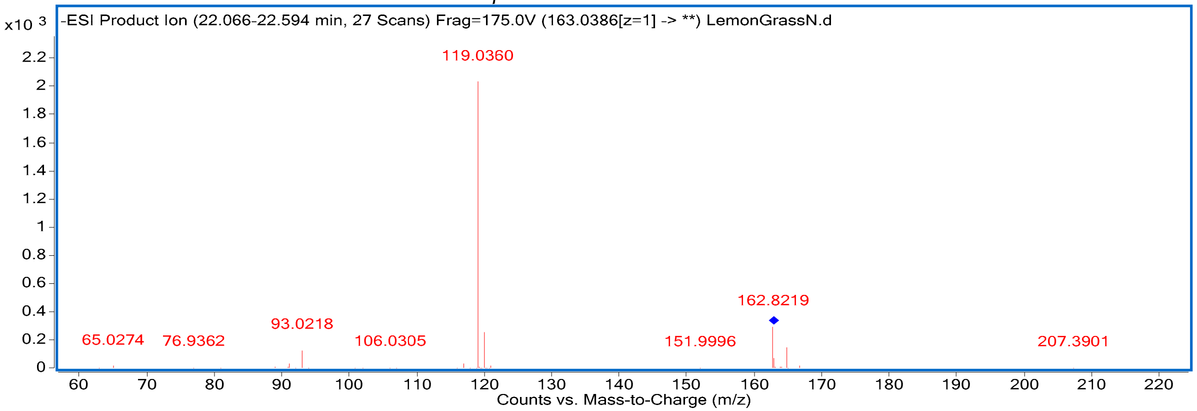

Chlorogenic acid

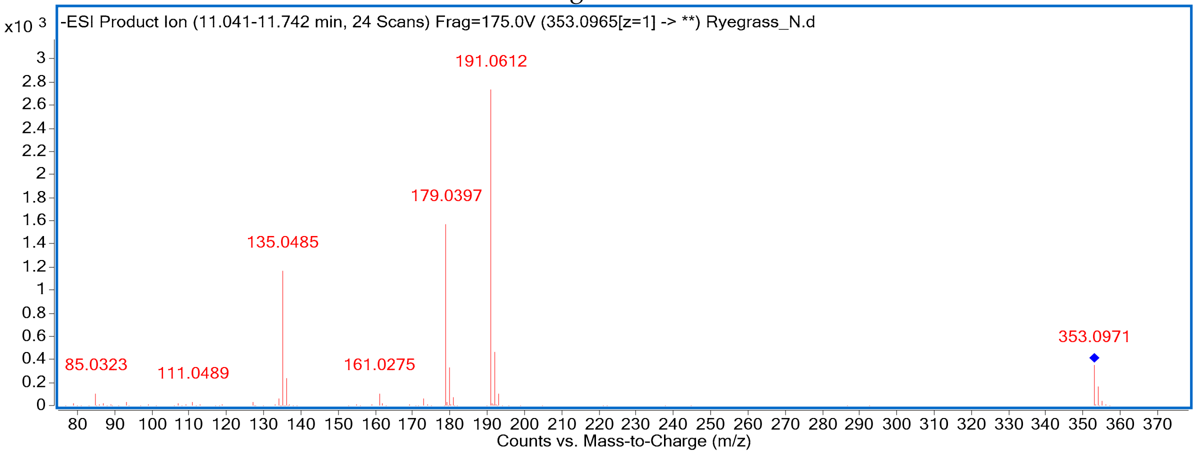

Figure S2. MS/MS spectra of some elected compounds in selected plants

Table S1. Quantification of abundant phenolic compounds (μg/g)

	**Compounds**	**Lemon grass**	**Rye**	**Chicory**	**Moringa**
1	Gallic Acid	11.01 ± 0.22	14.41 ± 1.72	23.59 ± 2.04	17.32 ± 1.79
2	Rosmarinic Acid		21.52 ± 1.65	72.36 ± 1.47	748.32 ± 25.82
3	*p*-hydroxybenzoic acid	36.13 ± 0.73	75.92 ± 5.67		
4	Cinnamic acid	91.38 ± 8.87		17.71 ± 1.18	31.32 ± 5.06
5	*p*-coumaric acid	244.63 ± 21.43		154.90 ± 12.62	17.61 ± 6.37
6	Ferulic acid	3.45 ± 0.61		189.41 ± 15.08	42.15 ± 4.96
7	3-Caffeoylquinic acid	341.52 ± 23.09	445.62 ± 31.52		103.93 ± 12.51
8	Caffeic acid	366.78 ± 14.77		245.67 ± 21.55	264.05 ± 11.54
9	Chicoric acid		15.82 ± 1.59	286.12 ± 31.27	
10	Sinapic acid	176.99 ± 4.76	51.05 ± 5.12	177.65 ± 5.43	
11	Protocatechuic acid	24.72 ± 1.94	63.14 ± 3.58		44.09 ± 3.04
12	Epicatechin	68.36 ± 0.44			168.82 ± 8.67
13	Procyanidin B2	44.62 ± 6.32			68.76 ± 4.12
14	Diosmin	16.48 ± 1.06	132.63 ± 11.06		9.51 ± 1.43
15	Pyrogallol	42.01 ± 77.74		111.5 ± 3.01	9.43± 1.31
16	Coumarin	17.3 ± 6.17			31.42 ± 2.89
17	3-Sinapoylquinic acid	96.32 ± 1.52	73.51 ± 3.83	70.88 ± 4.75	125.23 ± 19.42
18	Scopoletin			55.9 ± 5.57	13.73 ± 2.03
19	Umbelliferone		23.43 ±4.14		53.15 ± 6.82
20	Naringin	94.29 ± 0.93			11.03 ± 1.04
21	Carnosol				27.32 ± 1.94
22	Carnosic acid				32.53 ± 3.82

Table S2. The calculated binding energies of phenolic compounds

No.	compounds	Binding energies
1	Punicafolin	−12.16
2	Rutin	-11.14
3	Acarbose	-11.05
4	Procyanidin B2	-10.95
5	Myricitrin	-10.59
6	3-Feruloylquinic acid	-10.32
7	Taxifolin	-10.13
8	Diosmin	-9.84
9	quercitrin	-9.72
10	Chlorogenic acid	-9.62
11	Quercetin-3-O-arabinoside	-9.49
12	Naringin	-9.40
13	3-p-Coumaroylquinic acid	-9.35
14	Myricetin	-9.28
15	Quercetin	-6.95
16	Isorhamnetin	-6.68
17	Quinic acid	-6.65
18	Luteolin	-6.52
19	(-)-Epicatechin	-5.36
20	Hesperetin	-6.28
21	Gallic acid	-6.14
22	Quinic acid	-6.05
23	3-O-sinapoylquinic acid	-5.91
24	Diosmetin	-5.36
25	Naringenin	-5.33
26	p-Hydroxybenzoic acid	-5.30
27	Salicylic acid	-5.20
28	Caffeic acid	-5.08
29	*p*-coumaric acid	-4.80

30	Pyrogallol	-4.74
31	Chrysin	-4.19
32	Protocatechuic acid	-4.12
33	3-4-5-Trimethoxyflavone	-4.09
34	Cinnamic acid	-4.04
35	Coumarin	-3.93

Table S3. Predicted absorption and distribution of selected compounds.

	**Compounds**	Water solubility	Caco2 permeability	Intestinal absorption (human)	Skin Permeability	P-glycoprotein substrate	P-glycoprotein I inhibitor	P-glycoprotein II inhibitor	VDss (human)	Fraction unbound (human)	BBB permeability	CNS permeability
1	Gallic acid	-2.56	-0.081	43.374	-2.735	No	No	No	-1.855	0.617	-1.102	-3.74
2	Protocatechuic acid	-2.069	0.49	71.174	-2.727	No	No	No	-1.298	0.648	-0.683	-3.305
3	Protocatechuic acid 4-O-glucoside	-2.372	-0.733	15.09	-2.735	No	No	No	-0.728	0.725	-1.297	-4.056
4	Caffeic acid	-2.33	0.634	69.407	-2.722	No	No	No	-1.098	0.529	-0.647	-2.608
5	Piceatannol	-3.227	0.878	88.197	-2.735	Yes	No	No	0.438	0.237	-0.776	-2.257
6	Rosmarinic acid	-3.059	-0.937	32.516	-2.735	Yes	No	No	0.393	0.348	-1.378	-3.347
7	p-Hydroxybenzoic acid	-1.877	1.151	83.961	-2.723	No	No	No	-1.557	0.592	-0.334	-3.21
8	p-Coumaric acid	-2.378	1.21	93.494	-2.715	No	No	No	-1.151	0.428	-0.225	-2.418
9	p-Coumaric acid 4-O-glucoside	-2.393	-0.585	14.12	-2.735	No	No	No	-0.718	0.627	-1.076	-3.851
10	Kaempferol 3-O-	-2.866	-0.884	25.165	-2.735	Yes	No	No	1.295	0.28	-1.441	-3.955

	glucuronide											
11	Cinnamic acid	-2.608	1.717	94.833	-2.695	No	No	No	-1.051	0.38	0.446	-1.834
12	Coumarin	-1.517	1.649	97.344	-1.921	No	No	No	-0.143	0.367	-0.007	-1.926
13	Procyanidin trimer C1	-2.892	-1.826	60.469	-2.735	Yes	No	Yes	-0.086	0.378	-2.843	-4.855
14	Rosmanol	-3.606	1.015	93.407	-2.772	Yes	Yes	No	0.653	0.109	-0.581	-2.101
15	Carnosol	-4.116	0.572	91.206	-2.887	Yes	Yes	No	0.819	0.044	-0.096	-1.816
16	Carnosic acid	-3.224	0.803	99.03	-2.735	No	No	No	-1.027	0.048	-0.545	-1.998
17	Diosmin	-2.929	0.305	29.319	-2.735	Yes	No	No	1.428	0.105	-1.795	-4.836
18	Hesperidin	-3.014	0.505	31.481	-2.735	Yes	No	No	0.996	0.101	-1.715	-4.807
19	Mellein	-1.359	1.209	93.672	-2.563	No	No	No	0.166	0.476	-0.216	-2.284
20	Nobiletin	-4.949	1.306	98.921	-2.715	No	Yes	Yes	-0.281	0.179	-1.254	-3.142
21	Scopoletin	-2.504	1.184	95.277	-2.944	No	No	No	0.034	0.363	-0.299	-2.32
22	Secoisolariciresinol sesquilignan	-3.377	-0.237	61.217	-2.735	Yes	Yes	Yes	-0.412	0	-1.78	-3.892
23	Matairesinol	-3.685	1.115	93.527	-2.77	Yes	Yes	Yes	-0.265	0	-0.49	-3.071
24	Lariciresinol	-3.984	1.035	91.656	-2.818	Yes	No	No	-0.153	0	-0.834	-3.093
25	Lariciresinol-sesquilignan	-4.143	-0.432	75.194	-2.735	Yes	Yes	Yes	-0.31	0	-1.475	-3.662
26	Epicatechin	-3.117	-0.283	68.829	-2.735	Yes	No	No	1.027	0.235	-1.054	-3.298
27	Gallocatechin	-2.969	-0.375	54.128	-2.735	Yes	No	No	1.301	0.274	-1.377	-3.507
28	1,2-Diferuloylgentiobiose	-2.768	-0.85	29.394	-2.735	Yes	Yes	No	0.424	0.157	-2.011	-4.856
29	3,7-Dimethylquercetin	-1.991	-0.656	43.925	-2.735	Yes	No	No	0.034	0.612	-1.16	-3.693
30	3-Caffeoylquinic acid	-3.349	-0.351	84.383	-2.735	Yes	No	Yes	0.221	0.094	-1.408	-3.188
31	3-Feruloylquinic acid	-2.55	-0.564	44.764	-2.735	Yes	No	No	-1.303	0.392	-1.273	-3.943
32	3-p-Coumaroylquinic acid	-1.991	-0.656	43.925	-2.735	Yes	No	No	0.034	0.612	-1.16	-3.693
33	3-Sinapoylquinic acid	-2.547	-0.611	39.646	-2.735	Yes	No	No	-1.13	0.462	-1.396	-4.024
34	6''-O-Malonylgenistin	-2.88	-1.101	13.775	-2.735	Yes	No	No	0.208	0.259	-1.667	-3.971
35	6''-O-Malonylglycitin	-3.095	-0.762	30.329	-2.735	Yes	No	No	-0.083	0.145	-1.712	-4.259
36	Caffeic acid 4-O-glucuronide	-2.712	-0.801	0	-2.735	Yes	No	No	-0.447	0.654	-1.204	-4.15
37	Daidzein 7-O-	-2.854	-0.552	36.73	-2.735	Yes	No	No	-0.286	0.277	-1.145	-3.631

	glucuronide											
38	Equol 7-O-glucuronide	-2.963	-0.537	33.172	-2.735	Yes	No	No	-0.266	0.312	-0.836	-3.827
39	Esculin	-2.031	0.277	46.087	-2.749	Yes	No	No	-0.146	0.488	-1.255	-3.89
40	Ferulic acid	-2.817	0.176	93.685	-2.72	No	No	No	-1.367	0.343	-0.239	-2.612
41	Glycitein 7-O-glucuronide	-3.011	-0.612	25.769	-2.735	Yes	No	No	-0.209	0.151	-1.39	-4.021
42	Kaempferide	-3.238	0.326	79.898	-2.735	Yes	No	No	0.709	0.068	-0.954	-2.316
43	Medioresinol	-3.453	1.241	91.403	-2.777	Yes	Yes	Yes	0.21	0.021	-0.554	-3.037
44	Myricetin 3-O-rhamnoside	-2.892	-0.982	43.334	-2.735	Yes	No	No	1.552	0.182	-1.811	-4.376
45	Naringin	-2.919	-0.658	25.796	-2.735	Yes	No	No	0.619	0.159	-1.6	-4.773
46	Neoeriocitrin	-2.9	-0.855	16.143	-2.735	Yes	No	No	0.827	0.178	-1.836	-4.925
47	Procyanidin B2	-2.892	-1.225	66.749	-2.735	Yes	Yes	Yes	-0.158	0.309	-1.94	-3.983
48	Pyrogallol	-1.408	1.122	83.549	-2.751	No	No	No	0.13	0.712	-0.441	-3.252
49	Quercetin 3-O-xylosyl-glucuronide	-2.882	-1.274	39.35	-2.735	Yes	No	No	1.563	0.22	-2.051	-4.894
50	Rhoifolin	-2.862	-0.942	24.308	-2.735	Yes	No	No	1.14	0.152	-1.702	-4.798
51	Salvianolic acid B	-2.892	-1.57	9.902	-2.735	Yes	No	No	-0.078	0.214	-2.491	-4.187
52	Salvianolic acid C	-2.911	-1.397	41.447	-2.735	Yes	No	No	-0.4	0.069	-1.86	-3.394
53	Sinapic acid	-2.869	0.272	93.064	-2.725	Yes	No	No	-1.11	0.45	-0.247	-2.663
54	Taxifolin 4',7-diglucoside	-2.616	-1.164	0	-2.735	Yes	No	No	0.229	0.374	-1.959	-5.276
55	Theaflavin	-2.892	0.141	65.009	-2.735	Yes	Yes	Yes	0.679	0.2	-1.577	-4.33
56	Umbelliferone	-2.131	1.206	94.551	-2.6	No	No	No	0.032	0.432	-0.278	-2.741

Interpretation of results: If the Caco2 permeability value is higher than 0.90, a compound is considered to have a high Caco-2 permeability. A compound with a value of less than 30% is considered to be poorly absorbed in the human intestinal. If a compound has a value less than −2.5 is considered to have a low skin permeability. The compounds with values < −3 cannot penetrate the central nervous system while values > −2 are considered to penetrate the central nervous system. The compounds with values logBB < −1 are poorly distributed to the brain while values logBB > 0.3 are considered to cross the blood-brain barrier readily. VDss is considered low if below 0.71 L/kg (log VDss < −0.15) and high if above 2.81 L/kg (log VDss > 0.45).

Table S4. Pharmacokinetics properties of selected compounds

	Compounds	GI absorption	BBB permeant	Pgp substrate	CYP1A2 inhibitor	CYP2C19 inhibitor	CYP2C9 inhibitor	CYP2D6 inhibitor	CYP3A4 inhibitor	log Kp (cm/s)
1	Gallic acid	High	No	No	No	No	No	No	Yes	-6.84
2	Protocatechuic acid	High	No	No	No	No	No	No	Yes	-6.42
3	Protocatechuic acid 4-O-glucoside	Low	No	No	No	No	No	No	No	-9.21
4	Caffeic acid	High	No	No	No	No	No	No	No	-6.58
5	Piceatannol	High	No	No	Yes	No	Yes	No	Yes	-5.76
6	Rosmarinic acid	Low	No	No	No	No	No	No	No	-6.82
7	p-Hydroxybenzoic acid	High	Yes	No	No	No	No	No	No	-6.02
8	p-Coumaric acid	High	Yes	No	No	No	No	No	No	-6.26
9	p-Coumaric acid 4-O-glucoside	Low	No	No	No	No	No	No	No	-8.7
10	Kaempferol 3-O-glucuronide	Low	No	Yes	No	No	No	No	No	-8.44
11	Cinnamic acid	High	Yes	No	No	No	No	No	No	-5.69
12	Coumarin	High	Yes	No	Yes	No	No	No	No	-6.2
13	Procyanidin trimer C1	Low	No	Yes	No	No	No	No	No	-9.24
14	Rosmanol	High	No	Yes	No	No	No	Yes	No	-5.99
15	Carnosol	High	Yes	Yes	No	No	Yes	No	No	-5.21
16	Carnosic acid	High	No	No	No	No	Yes	No	No	-4.86
17	Diosmin	Low	No	Yes	No	No	No	No	No	-9.91
18	Hesperidin	Low	No	Yes	No	No	No	No	No	-10.12
19	Mellein	High	Yes	No	Yes	No	No	No	No	-5.65
20	Nobiletin	High	No	No	No	No	Yes	No	Yes	-6.62
21	Scopoletin	High	Yes	No	Yes	No	No	No	No	-6.39
22	Secoisolariciresinol-	Low	No	Yes	No	No	No	No	No	-7.8

	sesquilignan									
23	Matairesinol	High	No	No	No	No	No	Yes	Yes	-6.17
24	Lariciresinol	High	No	Yes	No	No	No	Yes	No	-6.79
25	Lariciresinol-sesquilignan	Low	No	No	No	No	No	No	Yes	-7.87
26	Epicatechin	High	No	Yes	No	No	No	No	No	-7.82
27	Gallocatechin	High	No	No	No	No	No	No	No	-8.17
28	1 2-Diferuloylgentiobiose	Low	No	Yes	No	No	No	No	No	-10.67
29	3 7-Dimethylquercetin	Low	No	No	No	No	No	No	No	-8.41
30	3-Caffeoylquinic acid	High	No	No	Yes	No	Yes	Yes	Yes	-6.16
31	3-Feruloylquinic acid	Low	No	No	No	No	No	No	No	-8.62
32	3-p-Coumaroylquinic acid	Low	No	No	No	No	No	No	No	-8.41
33	3-Sinapoylquinic acid	Low	No	Yes	No	No	No	No	No	-8.82
34	6-O-Malonylgenistin	Low	No	Yes	No	No	No	No	No	-9.05
35	6-O-Malonylglycitin	Low	No	Yes	No	No	No	No	No	-9.3
36	Caffeic acid 4-O-glucuronide	Low	No	No	No	No	No	No	No	-8.75
37	Daidzein 7-O-glucuronide	Low	No	No	No	No	No	No	No	-8.28
38	Equol 7-O-glucuronide	Low	No	Yes	No	No	No	No	No	-7.85
39	Esculin	Low	No	No	No	No	No	No	No	-8.81
40	Ferulic acid	High	Yes	No	No	No	No	No	No	-6.41
41	Glycitein 7-O-glucuronide	Low	No	No	No	No	No	No	No	-8.48
42	Kaempferide	High	No	No	Yes	No	No	Yes	Yes	-6.56
43	Medioresinol	High	No	Yes	No	No	No	Yes	No	-7.07
44	Myricetin 3-O-rhamnoside	Low	No	No	No	No	No	No	No	-8.77
45	Naringin	Low	No	Yes	No	No	No	No	No	-10.15

46	Neoeriocitrin	Low	No	Yes	No	No	No	No	No	-10.51
47	Procyanidin B2	Low	No	No	No	No	No	No	Yes	-8.15
48	Pyrogallol	High	Yes	No	No	No	No	No	Yes	-6.7
49	Quercetin 3-O-xylosyl-glucuronide	Low	No	Yes	No	No	No	No	No	-10.44
50	Rhoifolin	Low	No	Yes	No	No	No	No	No	-9.94
51	Salvianolic acid B	Low	No	No	No	No	No	No	No	-7.86
52	Salvianolic acid C	Low	No	No	No	No	Yes	No	No	-6.41
53	Sinapic acid	High	No	No	No	No	No	No	No	-6.63
54	Taxifolin 4 7-diglucoside	Low	No	No	No	No	No	No	No	-12.02
55	Theaflavin	Low	No	No	No	No	Yes	No	Yes	-8.05
56	Umbelliferone	High	Yes	No	Yes	No	No	No	No	-6.17

Table S5. Radar bioavailability properties of selected compounds

	Compounds	MW	Fraction Csp3	#Rotatable bonds	TPSA	XLOGP3	ESOL Log S
1	Gallic acid	170.12	0	1	97.99	0.7	-1.64
2	Protocatechuic acid	154.12	0	1	77.76	1.15	-1.86
3	Protocatechuic acid 4-O-glucoside	316.26	0.46	4	156.91	-1.38	-0.87
4	Caffeic acid	180.16	0	2	77.76	1.15	-1.89
5	Piceatannol	244.24	0	2	80.92	2.86	-3.52
6	Rosmarinic acid	360.31	0.11	7	144.52	2.36	-3.44
7	p-Hydroxybenzoic acid	138.12	0	1	57.53	1.58	-2.07
8	p-Coumaric acid	164.16	0	2	57.53	1.46	-2.02
9	p-Coumaric acid 4-O-glucoside	326.3	0.4	5	136.68	-0.57	-1.37

10	Kaempferol 3-O-glucuronide	462.36	0.24	4	207.35	0.96	-3.41
11	Cinnamic acid	148.16	0	2	37.3	2.13	-2.37
12	Coumarin	146.14	0	0	30.21	1.39	-2.29
13	Procyanidin trimer C1	866.77	0.2	5	331.14	3.31	-7.39
14	Rosmanol	346.42	0.65	1	86.99	3.41	-4.25
15	Carnosol	330.42	0.65	1	66.76	4.38	-4.77
16	Carnosic acid	332.43	0.65	2	77.76	4.89	-5.03
17	Diosmin	608.54	0.46	7	238.2	0.14	-3.51
18	Hesperidin	610.56	0.54	7	234.29	-0.14	-3.28
19	Mellein	178.18	0.3	0	46.53	2.44	-2.82
20	Nobiletin	402.39	0.29	7	85.59	3.01	-4.18
21	Scopoletin	192.17	0.1	1	59.67	1.53	-2.46
22	Secoisolariciresinol-sesquilignan	558.62	0.4	15	158.3	2.69	-4.34
23	Matairesinol	358.39	0.35	6	85.22	3.26	-4.06
24	Lariciresinol	360.4	0.4	6	88.38	2.4	-3.53
25	Lariciresinol-sesquilignan	556.6	0.4	12	147.3	2.57	-4.45
26	Epicatechin	290.27	0.2	1	110.38	0.36	-2.22
27	Gallocatechin	306.27	0.2	1	130.61	0	-2.08
28	1 2-Diferuloylgentiobiose	694.63	0.44	14	260.59	-0.19	-3.28
29	3 7-Dimethylquercetin	338.31	0.38	5	144.52	-0.07	-1.75
30	3-Caffeoylquinic acid	330.29	0.12	3	109.36	3.04	-4.1
31	3-Feruloylquinic acid	368.34	0.41	6	153.75	-0.1	-1.84
32	3-p-Coumaroylquinic acid	338.31	0.38	5	144.52	-0.07	-1.75
33	3-Sinapoylquinic acid	398.36	0.44	7	162.98	-0.13	-1.92
34	6-O-Malonylgenistin	518.42	0.29	8	213.42	0.58	-3.21
35	6-O-Malonylglycitin	532.45	0.32	9	202.42	0.35	-3.08
36	Caffeic acid 4-O-glucuronide	356.28	0.33	5	173.98	-0.39	-1.65
37	Daidzein 7-O-glucuronide	430.36	0.24	4	166.89	0.91	-3.2
38	Equol 7-O-glucuronide	418.39	0.38	4	145.91	1.41	-3.35
39	Esculin	340.28	0.4	3	149.82	-0.61	-1.68

40	Ferulic acid	194.18	0.1	3	66.76	1.51	-2.11
41	Glycitein 7-O-glucuronide	460.39	0.27	5	176.12	0.88	-3.28
42	Kaempferide	300.26	0.06	2	100.13	2.22	-3.51
43	Medioresinol	388.41	0.43	5	86.61	2.25	-3.65
44	Myricetin 3-O-rhamnoside	464.38	0.29	3	210.51	0.51	-3.2
45	Naringin	580.53	0.52	6	225.06	-0.44	-2.98
46	Neoeriocitrin	596.53	0.52	6	245.29	-0.8	-2.85
47	Procyanidin B2	578.52	0.2	3	220.76	2.37	-5.14
48	Pyrogallol	126.11	0	0	60.69	0.52	-1.44
49	Quercetin 3-O-xylosyl-glucuronide	610.47	0.38	7	286.5	-0.58	-3.07
50	Rhoifolin	578.52	0.44	6	228.97	-0.16	-3.22
51	Salvianolic acid B	718.61	0.17	14	278.04	3.98	-6.22
52	Salvianolic acid C	492.43	0.08	8	177.89	4.07	-5.36
53	Sinapic acid	224.21	0.18	4	75.99	1.46	-2.16
54	Taxifolin 4 7-diglucoside	628.53	0.52	7	285.75	-2.66	-1.8
55	Theaflavin	564.49	0.21	2	217.6	2.38	-5.12
56	Umbelliferone	162.14	0	0	50.44	1.58	-2.46

(Lipophilicity: XLOGP3 between -0.7 and +5.0, size: MW between 150 and 500 g/mol, polarity: TPSA between 20 and 130Å2, solubility: log S not higher than 6, saturation: fraction of carbons in the sp3 hybridization not less than 0.25, and flexibility: no more than nine rotatable bonds; topological polar surface area (TPSA), Only quinic acid, satisfied all parameters for drug-likeness (oral bioavailability)

Table S6. Metabolism and excretion of selected compounds

	**Compounds**	CYP2D6 substrate	CYP3A4 substrate	CYP1A2 inhibitior	CYP2C19 inhibitior	CYP2C9 inhibitior	CYP2D6 inhibitior	CYP3A4 inhibitior	Total Clearance	Renal OCT2 substrate
1	Gallic acid	No	No	No	No	No	No	No	0.518	No
2	Protocatechuic acid	No	No	No	No	No	No	No	0.551	No
3	Protocatechuic acid 4-O-glucoside	No	No	No	No	No	No	No	0.552	No
4	Caffeic acid	No	No	No	No	No	No	No	0.508	No
5	Piceatannol	No	Yes	Yes	Yes	No	Yes	No	0.004	No
6	Rosmarinic acid	No	No	No	No	No	No	No	0.25	No
7	p-Hydroxybenzoic acid	No	No	No	No	No	No	No	0.593	No
8	p-Coumaric acid	No	No	No	No	No	No	No	0.662	No
9	p-Coumaric acid 4-O-glucoside	No	No	No	No	No	No	No	0.278	No
10	Kaempferol 3-O-glucuronide	No	No	No	No	No	No	No	0.503	No
11	Cinnamic acid	No	No	No	No	No	No	No	0.781	No
12	Coumarin	No	No	Yes	No	No	No	No	0.97	No
13	Procyanidin trimer C1	No	No	No	No	No	No	No	-3.326	No
14	Rosmanol	No	No	No	No	No	No	No	0.289	No
15	Carnosol	No	Yes	No	Yes	No	No	No	0.28	No
16	Carnosic acid	No	No	No	No	No	No	No	0.379	No
17	Diosmin	No	No	No	No	No	No	No	-0.113	No
18	Hesperidin	No	No	No	No	No	No	No	0.211	No
19	Mellein	No	No	No	No	No	No	No	0.584	No
20	Nobiletin	No	Yes	Yes	Yes	Yes	No	Yes	0.789	No
21	Scopoletin	No	No	Yes	No	No	No	No	0.73	No

22	Secoisolariciresinol sesquilignan	No	Yes	No	No	No	No	Yes	0.202	No
23	Matairesinol	No	Yes	Yes	Yes	Yes	No	Yes	0.147	No
24	Lariciresinol	No	Yes	No	Yes	No	No	Yes	0.139	No
25	Lariciresinol-sesquilignan	No	Yes	No	No	Yes	No	Yes	0.094	No
26	Epicatechin	No	No	No	No	No	No	No	0.183	No
27	Gallocatechin	No	No	No	No	No	No	No	0.328	No
28	1,2-Diferuloylgentiobiose	No	No	No	No	No	No	No	0.463	No
29	3,7-Dimethylquercetin	No	No	No	No	No	No	No	0.453	No
30	3-Caffeoylquinic acid	No	No	Yes	Yes	No	No	No	0.531	No
31	3-Feruloylquinic acid	No	No	No	No	No	No	No	0.393	No
32	3-p-Coumaroylquinic acid	No	No	No	No	No	No	No	0.453	No
33	3-Sinapoylquinic acid	No	No	No	No	No	No	No	0.727	No
34	6''-O-Malonylgenistin	No	No	No	No	No	No	No	0.226	No
35	6''-O-Malonylglycitin	No	No	No	No	No	No	No	0.277	No
36	Caffeic acid 4-O-glucuronide	No	No	No	No	No	No	No	0.194	No
37	Daidzein 7-O-glucuronide	No	No	No	No	No	No	No	0.067	No
38	Equol 7-O-glucuronide	No	No	No	No	No	No	No	0.529	No
39	Esculin	No	No	No	No	No	No	No	0.651	No
40	Ferulic acid	No	No	No	No	No	No	No	0.623	No
41	Glycitein 7-O-glucuronide	No	No	No	No	No	No	No	0.062	No
42	Kaempferide	No	No	Yes	Yes	Yes	No	No	0.569	No

43	Medioresinol	No	Yes	No	Yes	Yes	No	Yes	0.05	No
44	Myricetin 3-O-rhamnoside	No	No	No	No	No	No	No	0.303	No
45	Naringin	No	No	No	No	No	No	No	0.318	No
46	Neoeriocitrin	No	No	No	No	No	No	No	0.125	No
47	Procyanidin B2	No	No	No	No	No	No	No	-0.085	Yes
48	Pyrogallol	No	No	No	No	No	No	No	0.104	No
49	Quercetin 3-O-xylosyl-glucuronide	No	No	No	No	No	No	No	-0.403	No
50	Rhoifolin	No	No	No	No	No	No	No	-0.005	No
51	Salvianolic acid B	No	No	No	No	No	No	No	-0.609	No
52	Salvianolic acid C	No	Yes	No	No	No	No	No	0.132	No
53	Sinapic acid	No	No	No	No	No	No	No	0.718	No
54	Taxifolin 4',7-diglucoside	No	No	No	No	No	No	No	0.168	No
55	Theaflavin	No	No	No	No	No	No	No	0.081	No
56	Umbelliferone	No	No	Yes	No	No	No	No	0.706	No

Table S7 Predicted toxicity of abundant phenolic compounds

	**Compounds**	AMES toxicity	Max. tolerated dose (human)	hERG I inhibitor	hERG II inhibitor	Oral Rat Acute Toxicity (LD50)	Oral Rat Chronic Toxicity (LOAEL)	Hepatotoxicity	Skin Sensitisation	<i>T.Pyriformis</i> toxicity	Minnow toxicity
1	Gallic acid	No	0.7	No	No	2.218	3.06	No	No	0.285	3.188
2	Protocatechuic acid	No	0.814	No	No	2.423	2.021	No	No	0.273	2.451
3	Protocatechuic acid 4-O-glucoside	No	0.592	No	No	2.576	3.661	No	No	0.285	5.526
4	Caffeic acid	No	1.145	No	No	2.383	2.092	No	No	0.293	2.246
5	Piceatannol	No	0.338	No	No	2.529	0.953	No	No	0.563	2.161
6	Rosmarinic acid	No	0.152	No	No	2.811	2.907	No	No	0.302	2.698
7	p-Hydroxybenzoic acid	No	0.846	No	No	2.255	2.483	No	No	0.268	1.812
8	p-Coumaric acid	No	1.111	No	No	2.155	2.534	No	No	0.319	1.607
9	p-Coumaric acid 4-O-glucoside	No	0.492	No	No	2.414	3.751	No	No	0.285	3.798
10	Kaempferol 3-O-glucuronide	No	0.46	No	No	2.513	4.641	No	No	0.285	6.898
11	Cinnamic acid	No	1.11	No	No	2.094	2.651	No	No	0.247	1.719
12	Coumarin	No	0.435	No	No	2.112	1.903	No	No	0.365	1.555
13	Procyanidin trimer C1	No	0.438	No	Yes	2.482	7.463	No	No	0.285	14.258
14	Rosmanol	No	0.331	No	Yes	1.977	2.547	Yes	No	0.329	0.285
15	Carnosol	No	0.227	No	No	2.192	1.909	No	No	0.405	-0.636
16	Carnosic acid	No	0.345	No	No	2.891	1.972	No	No	0.285	-0.627

17	Diosmin	No	0.565	No	Yes	2.512	3.343	No	No	0.285	5.348
18	Hesperidin	No	0.525	No	Yes	2.506	3.167	No	No	0.285	7.131
19	Mellein	No	0.205	No	No	2.022	2.137	Yes	No	0.857	1.865
20	Nobiletin	No	0.443	No	No	2.459	0.82	No	No	0.315	0.686
21	Scopoletin	No	0.614	No	No	1.95	1.378	No	No	0.516	1.614
22	Secoisolariciresinol sesquilignan	No	0.407	No	Yes	2.762	3.576	No	No	0.285	1.742
23	Matairesinol	No	-0.164	No	Yes	1.935	2.151	No	No	0.454	0.44
24	Lariciresinol	No	-0.099	No	Yes	2	2.226	No	No	0.427	1.316
25	Lariciresinol-sesquilignan	No	0.114	No	Yes	2.444	2.416	No	No	0.285	2.135
26	Epicatechin	No	0.438	No	No	2.428	2.5	No	No	0.347	3.585
27	Gallocatechin	No	0.506	No	No	2.492	2.927	No	No	0.286	4.235
28	1,2-Diferuloylgentiobiose	No	0.069	No	No	2.587	4.869	No	No	0.285	8.945
29	3,7-Dimethylquercetin	No	-0.09	No	No	1.737	2.508	No	No	0.285	4.611
30	3-Caffeoylquinic acid	No	0.289	No	No	2.351	2.646	No	No	0.343	1.712
31	3-Feruloylquinic acid	No	1.285	No	No	2.025	4.485	No	No	0.285	4.876
32	3-p-Coumaroylquinic acid	No	-0.09	No	No	1.737	2.508	No	No	0.285	4.611
33	3-Sinapoylquinic acid	No	0.862	No	No	1.994	3.807	No	No	0.285	5.242
34	6''-O-Malonylgenistin	No	0.507	No	No	2.547	3.798	No	No	0.285	5.927
35	6''-O-Malonylglycitin	No	0.558	No	No	2.595	3.324	No	No	0.285	3.941

36	Caffeic acid 4-O-glucuronide	No	0.656	No	No	2.462	4.259	Yes	No	0.285	4.797
37	Daidzein 7-O-glucuronide	No	0.663	No	No	2.698	4.82	No	No	0.285	3.869
38	Equol 7-O-glucuronide	No	0.295	No	No	2.801	4.598	No	No	0.285	3.333
39	Esculin	No	0.799	No	No	2.299	4.157	Yes	No	0.285	4.925
40	Ferulic acid	No	1.082	No	No	2.282	2.065	No	No	0.271	1.825
41	Glycitein 7-O-glucuronide	No	0.603	No	No	2.733	3.961	No	No	0.285	3.2
42	Kaempferide	No	0.42	No	No	2.338	2.271	No	No	0.336	1.737
43	Medioresinol	No	-0.874	No	No	2.149	2.097	No	No	0.355	1.253
44	Myricetin 3-O-rhamnoside	No	0.454	No	Yes	2.537	3.386	No	No	0.285	5.997
45	Naringin	No	0.43	No	Yes	2.495	4.202	No	No	0.285	6.042
46	Neoeriocitrin	No	0.475	No	Yes	2.487	4.385	No	No	0.285	6.975
47	Procyanidin B2	No	0.438	No	Yes	2.482	4.349	No	No	0.285	8.704
48	Pyrogallol	No	-0.269	No	No	2.049	2.374	No	No	0.127	2.734
49	Quercetin 3-O-xylosyl-glucuronide	No	0.456	No	Yes	2.484	4.797	No	No	0.285	12.724
50	Rhoifolin	No	0.492	No	Yes	2.498	4.443	No	No	0.285	3.865
51	Salvianolic acid B	No	0.439	No	No	2.482	5.46	No	No	0.285	4.64
52	Salvianolic acid C	No	0.499	No	No	2.592	3.175	No	No	0.285	-0.392
53	Sinapic acid	No	1.193	No	No	2.24	2.324	No	No	0.262	2.18
54	Taxifolin 4',7-diglucoside	No	0.18	No	Yes	2.485	5.473	No	No	0.285	12.41
55	Theaflavin	No	0.439	No	Yes	2.505	4.008	No	No	0.285	6.902
56	Umbelliferone	No	0.689	No	No	2.047	1.751	Yes	No	0.546	1.714

Explanations

Minnow toxicity

LC50 values below 0.5mM (log LC50 < −0.3) are regarded as high acute toxicity.

***T. Pyriformis* toxicity**

pIGC50 (negative logarithm of the concentration required to inhibit 50% growth in log µg/L) is considered, with a value > −0.5 log µg/L is considered toxic.

AMES toxicity: if the AMES value is positive, then the compound will be mutagenic.

Maximum tolerated dose: a value less than is equal to 0.477 log(mg/kg/day) is considered low and high if greater than 0.477 log(mg/kg/day)
